# Cytoplasmic retention of the DNA/RNA-binding protein FUS ameliorates organ fibrosis in mice

**DOI:** 10.1172/JCI175158

**Published:** 2024-03-15

**Authors:** Manuel Chiusa, Youngmin A. Lee, Ming-Zhi Zhang, Raymond C. Harris, Taylor Sherrill, Volkhard Lindner, Craig R. Brooks, Gang Yu, Agnes B. Fogo, Charles R. Flynn, Jozef Zienkiewicz, Jacek Hawiger, Roy Zent, Ambra Pozzi

**Affiliations:** 1 Department of Medicine, Division of Nephrology and Hypertension, and; 2 Department of Surgery, Vanderbilt University Medical Center, Nashville, Tennessee, USA.; 3 Department of Veterans Affairs, Nashville, Tennessee, USA.; 4 Department of Medicine, Division of Allergy, Pulmonary and Critical Care Medicine, Vanderbilt University Medical Center, Nashville, Tennessee, USA.; 5 Center for Molecular Medicine, Maine Health Institute for Research, Scarborough, Maine, USA.; 6 Department of Neuroscience, Peter O’Donnell Jr. Brain Institute, University of Texas Southwestern Medical Center, Dallas, Texas, USA.; 7 Department of Pathology, Microbiology and Immunology, Vanderbilt University Medical Center, Nashville, Tennessee, USA.

**Keywords:** Hepatology, Nephrology, Extracellular matrix, Growth factors, Integrins

## Abstract

Uncontrolled accumulation of extracellular matrix leads to tissue fibrosis and loss of organ function. We previously demonstrated in vitro that the DNA/RNA-binding protein fused in sarcoma (FUS) promotes fibrotic responses by translocating to the nucleus, where it initiates collagen gene transcription. However, it is still not known whether FUS is profibrotic in vivo and whether preventing its nuclear translocation might inhibit development of fibrosis following injury. We now demonstrate that levels of nuclear FUS are significantly increased in mouse models of kidney and liver fibrosis. To evaluate the direct role of FUS nuclear translocation in fibrosis, we used mice that carry a mutation in the FUS nuclear localization sequence (FUSR521G) and the cell-penetrating peptide CP-FUS-NLS that we previously showed inhibits FUS nuclear translocation in vitro. We provide evidence that *FUSR521G* mice or CP-FUS-NLS–treated mice showed reduced nuclear FUS and fibrosis following injury. Finally, differential gene expression analysis and immunohistochemistry of tissues from individuals with focal segmental glomerulosclerosis or nonalcoholic steatohepatitis revealed significant upregulation of FUS and/or collagen genes and FUS protein nuclear localization in diseased organs. These results demonstrate that injury-induced nuclear translocation of FUS contributes to fibrosis and highlight CP-FUS-NLS as a promising therapeutic option for organ fibrosis.

## Abstract 2

Prevention of the nuclear translocation of Fused in Sarcoma alleviates organ fibrosis in mouse models.

## Graphical abstract

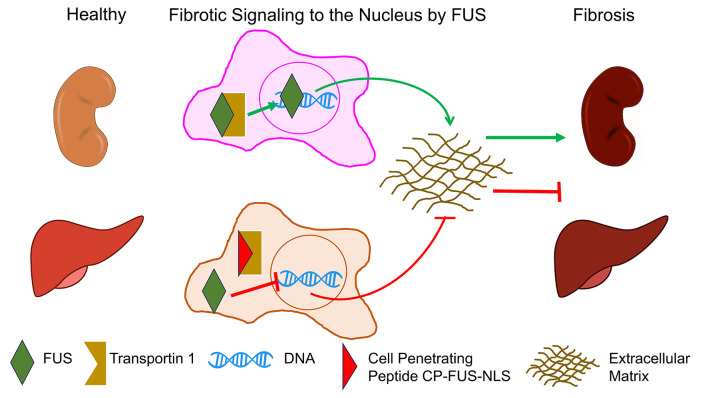

## Introduction

Organ fibrosis is characterized by excessive deposition of extracellular matrix (ECM) components (mainly collagen) within an injured organ that leads to the disruption of normal tissue architecture and loss of organ function. At present, therapeutic options for fibrosis are limited. A major obstacle to the development of antifibrotic therapies is that fibrosis is a multicellular event that requires physical as well as humoral crosstalk among different cell types within an injured organ. In addition, ECM homeostasis is regulated by several factors, including cellular receptors such as integrins and receptor tyrosine kinases (RTKs) (1, 2). We previously showed that the collagen-binding receptor integrin α1β1 is a negative regulator of fibrotic responses in kidney cells (3). A mechanism whereby this receptor downregulates collagen synthesis is by negatively modulating the phosphorylation/activation of the epidermal growth factor receptor (EGFR) via recruitment of the T cell protein tyrosine phosphatase PTPN2 (4).

EGFR activation has been implicated in the development of fibrosis in several organs, including kidney and liver. In the kidney, prolonged and/or aberrant EGFR signaling is a key determinant of progressive fibrotic injury (5), and glomerular activation of EGFR is a key step in the development of rapid progressive glomerulonephritis in both humans and mice (6). In the liver, EGF is upregulated in a rat model of liver fibrogenesis and in human cirrhotic liver tissues (7, 8), and a polymorphism in the human EGF gene that leads to increased EGF expression is associated with increased fibrosis and cirrhosis progression in patients with chronic hepatitis C (9).

Although EGFR plays a detrimental role in organ fibrosis, the use of EGFR inhibitors in humans leads to severe side effects, including skin toxicity (10). In addition, blocking EGFR might affect liver regeneration, as this process requires a functional EGF/EGFR axis in hepatocytes (7). Thus, a better understanding of selective fibrotic signaling activated downstream of the EGFR might lead to the development of safer and better-tolerated therapies for EGFR-mediated organ fibrosis.

We recently showed that a mechanism whereby EGFR regulates ECM production and in turn fibrotic responses is by promoting the nuclear translocation of the DNA/RNA-binding protein fused in sarcoma (FUS) in the mesangial cells of the kidney (11). Mechanistically, EGFR phosphorylates FUS on tyrosines 6 and 296, thus promoting FUS nuclear translocation. Nuclear FUS binds to the bidirectional promoter of collagen IV α1 and α2 chains, commencing its gene transcription (11). Consistent with this finding, mesangial cells lacking integrin α1β1 show not only increased baseline EGFR activation but also increased levels of nuclear FUS (11). Further corroborating a fibrotic role of FUS, we showed that mutating tyrosines 6 and 296 (FUS-Y6/296F) prevented EGF-induced FUS nuclear translocation and collagen IV production in mesangial cells in vitro (11). Consistent with this finding, downregulation of FUS in cardiac fibroblasts prevented PAX3 stability and, in turn, angiotensin II–induced collagen production (12). Further reinforcing the concept that prevention of FUS nuclear translocation has antifibrotic potential, patients affected by amyotrophic lateral sclerosis carrying mutations of FUS known to confer cytoplasmic gain of function have a decreased amount of collagen IV in skin, urine, and plasma (13–15). Finally, we showed that treatment of mesangial cells with the cell-penetrating peptide CP-FUS-NLS, which binds to transportin 1 (a nuclear import adaptor protein also known as karyopherin β2 or importin β2), inhibited FUS nuclear translocation and collagen IV transcription (11).

Although preventing FUS nuclear translocation inhibits collagen transcription, the fibrotic action of FUS has been investigated primarily in cell culture systems. Whether FUS has a role in governing fibrotic responses in vivo and whether preventing its nuclear translocation reduces fibrosis in vivo have not been investigated. We provide genetic evidence that mice expressing FUS carrying a mutation in the nuclear localization sequence (NLS) are protected from the development of fibrosis following kidney injury. Importantly, we provide pharmacologic evidence that mice treated with CP-FUS-NLS developed significantly less fibrosis following both kidney- and liver-induced injury. Our study shows that nuclear translocation of FUS in response to kidney and liver injury is linked to the promotion of fibrosis. As inhibition of FUS nuclear translocation ameliorates fibrotic responses in vivo, we propose that nuclear translocation of FUS can be viewed as a targetable step in organ fibrosis.

## Results

### Genetic or pharmacologic inhibition of EGFR kinase activity ameliorates adriamycin-induced kidney injury.

**Figure 1:**
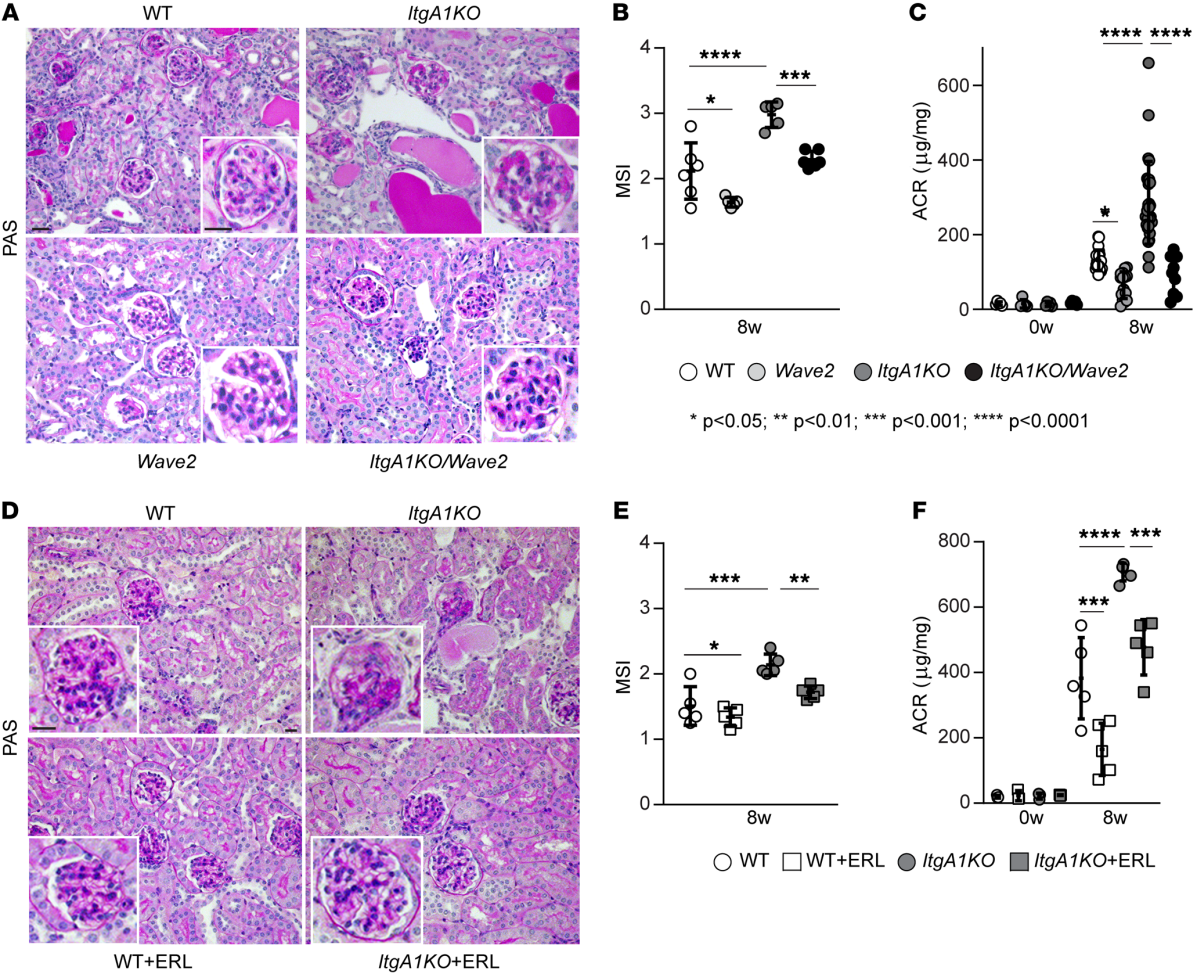
EGFR contributes to ADR-induced glomerular injury. (**A**) Representative images of periodic acid–Schiff–stained kidney sections from WT, *ItgA1KO*, *Wave2*, and *ItgA1KO/Wave2* mice treated with ADR for 8 weeks. Scale bars: 20 μm. (**B**) Mesangial sclerosis index (MSI) of kidneys shown in **A** was evaluated and scored as described in Methods. Values are the mean ± SD, and symbols represent individual kidneys (*n* = 6 WT, *n* = 5 *ItgA1KO*, *n* = 5 *Wave2*, *n* = 6 *ItgA1KO/Wave2*, with ~20 glomeruli per kidney evaluated). (**C**) Albumin/creatinine ratio (ACR) was evaluated at baseline (*n* = 5 WT, *n* = 5 *ItgA1KO*, *n* = 5 *Wave2*, *n* = 5 *ItgA1KO/Wave2*) and 8 weeks (*n* = 22 WT, *n* = 29 *ItgA1KO*, *n* = 15 *Wave2*, *n* = 10 *ItgA1KO/Wave2*) after ADR injection. Symbols represent individual mice. (**D**) Representative images of periodic acid–Schiff–stained kidneys from WT and *ItgA1KO* mice treated with ADR for 8 weeks and treated with vehicle or erlotinib (ERL). Scale bars: 20 μm. (**E**) MSI of kidneys shown in **D** was evaluated and scored as described in Methods. Values are the mean ± SD, and symbols represent individual kidneys (*n* = 5 WT, *n* = 5 *ItgA1KO*, *n* = 5 WT+ERL, *n* = 5 *ItgA1KO*+ERL, with ~20 glomeruli per kidney). (**F**) ACR was evaluated at baseline (*n* = 3 WT, *n* = 3 *ItgA1KO*, *n* = 3 WT+ERL, *n* = 3 *ItgA1KO*+ERL) and 8 weeks (*n* = 5 WT, *n* = 5 *ItgA1KO*, *n* = 5 WT+ERL, *n* = 5 *ItgA1KO*+ERL) after ADR injection. Symbols represent individual mice. Statistical analysis: 1-way ANOVA followed by Dunnett’s multiple-comparison test (**B**, **C**, **E**, and **F**).

We previously showed that EGFR promotes fibrotic responses in mesangial cells within the glomerulus of the kidney by phosphorylating FUS, which promotes its nuclear translocation and the commencement of collagen transcription (11). We also showed that fibrotic FUS and EGFR are activated in mesangial cells and in mice lacking integrin α1β1 (*ItgA1KO*) (4, 11), a negative regulator of EGFR phosphorylation/activation. To define the role of EGFR-mediated FUS nuclear translocation in glomerular injury, we induced glomerular injury in mice via administration of adriamycin (ADR), a well-established model of focal segmental glomerulosclerosis (16). We evaluated the degree of glomerular injury in ADR-treated BALB/c wild-type (WT) and *ItgA1KO* mice, as well as *Wave2* mice and *Wave2* mice crossed onto the *ItgA1KO* background (*ItgA1KO/Wave2*) (genetic approach). *Wave2* mice possess a single-nucleotide mutation in the gene encoding EGFR, resulting in over 90% global reduction of EGFR tyrosine kinase activity (17). Renal injury was assessed by histologic examination 8 weeks after ADR treatment. As previously shown (3), both ADR-treated WT and *ItgA1KO* mice had severe mesangial expansion and well-developed glomerulosclerosis (Figure 1, A and B). However, the degree of injury was significantly higher in the *ItgA1KO* mice (Figure 1, A and B). Consistent with the glomerular injury, injured *ItgA1KO* mice had a significantly increased urinary albumin/creatinine ratio compared with WT mice (Figure 1C). Analysis of *Wave2* and *ItgA1KO/Wave2* mice revealed significantly reduced glomerular injury (Figure 1, A and B) and albuminuria (Figure 1C) in comparison with injured WT and *ItgA1KO* mice, respectively.

To further corroborate the role of EGFR in mediating glomerular injury, we investigated the degree of injury in ADR-treated WT and *ItgA1KO* mice treated with the selective EGFR inhibitor erlotinib for 8 weeks (pharmacologic approach). This inhibitor significantly reduced both glomerular injury and albuminuria in both WT and *ItgA1KO* (Figure 1, D–F), suggesting that activation of EGFR plays a deleterious role in ADR-mediated kidney injury.

### Genetic or pharmacologic inhibition of EGFR kinase activity decreases ADR-induced kidney fibrosis.

**Figure 2:**
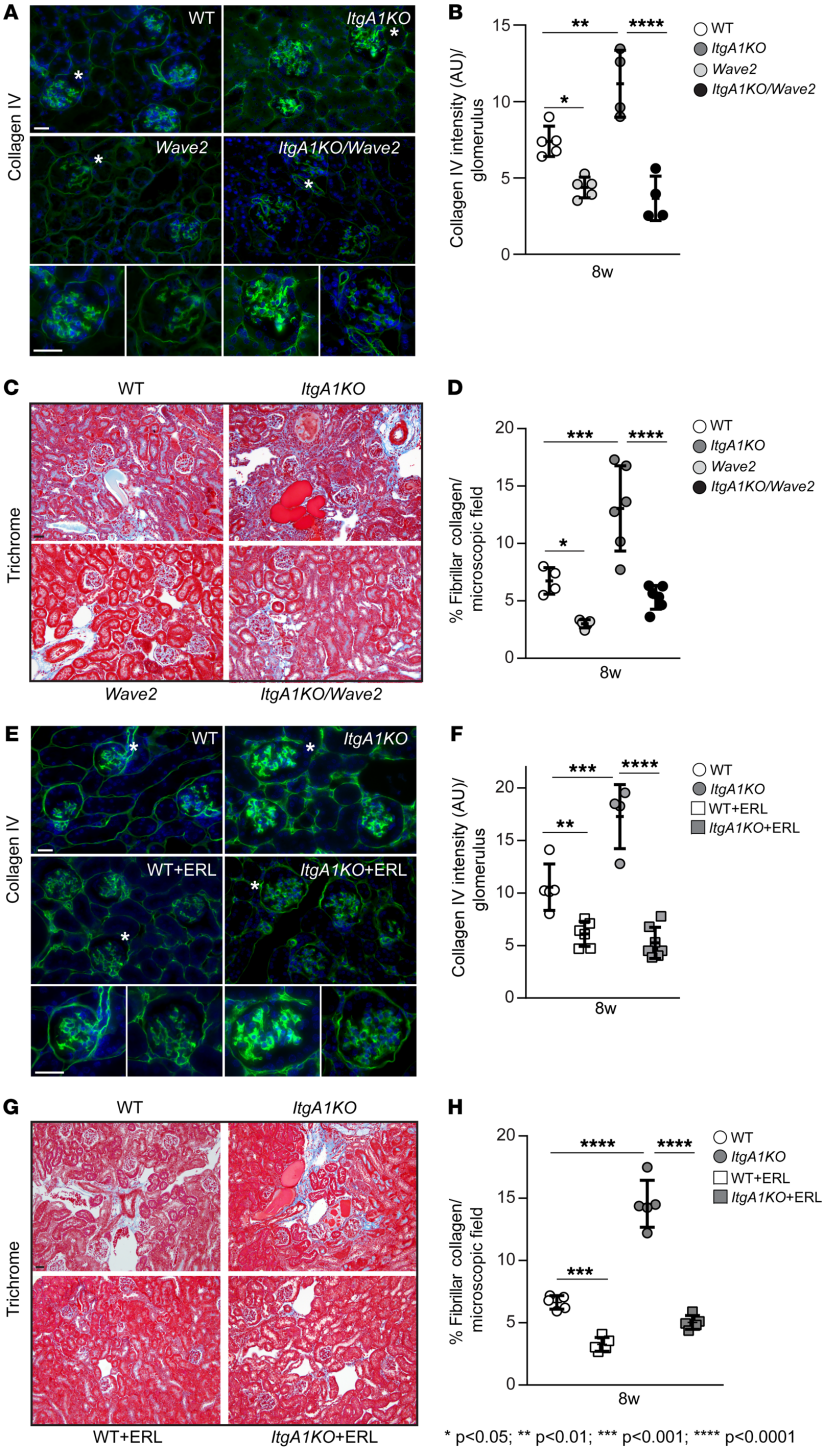
EGFR contributes to ADR-induced glomerulosclerosis. (**A**, **C**, **E**, and **G**) Representative light microscopy of collagen IV–stained (**A** and **E**) or Masson’s trichrome–stained (**C** and **G**) kidney sections from the mice indicated 8 weeks after ADR injection. Asterisks indicate single glomeruli. Scale bars: 25 μm. (**B**, **D**, **F**, and **H**) The amount of collagen IV per glomerulus (**B**, *n* = 5 WT, *n* = 4 *ItgA1KO*, *n* = 5 *Wave2*, *n* = 4 *ItgA1KO/Wave2*; **F**, *n* = 5 WT, *n* = 4 *ItgA1KO*, *n* = 6 WT+ERL, *n* = 7 *ItgA1KO*+ERL) or fibrillar collagen per microscopic field (**D**, *n* = 4 WT, *n* = 6 *ItgA1KO*, *n* = 5 *Wave2*, *n* = 6 *ItgA1KO/Wave2*; **H**, *n* = 5 WT, *n* = 5 *ItgA1KO*, *n* = 5 WT+ERL, *n* = 5 *ItgA1KO*+ERL) was evaluated using ImageJ as described in Methods. Values are the mean ± SD, and symbols represent individual kidneys (with an average of at least 12 glomeruli per kidney or three ×20 microscopic fields per kidney). Statistical analysis: 1-way ANOVA followed by Dunnett’s multiple-comparison test (**B**, **D**, **F**, and **H**).

One of the key features of glomerulosclerosis is increased collagen deposition. Loss of integrin α1β1 leads to increased collagen production via EGFR activation (3). Thus, we investigated the levels of nonfibrillar collagen IV and fibrillar collagens by immunohistochemistry and Masson’s trichrome staining, respectively. Both techniques clearly revealed more collagen staining in the glomeruli of injured *ItgA1KO* mice compared with WT mice (Figure 2, A–H). Collagen accumulation was significantly decreased in *Wave*2 and *ItgA1KO/Wave2* mice (Figure 2, A–D). Similarly, treatment of *ItgA1KO* mice with erlotinib resulted in greater reductions of both nonfibrillar and fibrillar collagen deposition compared with erlotinib-treated WT mice (Figure 2, E–H).

### Genetic or pharmacologic inhibition of EGFR kinase activity decreases glomerular nuclear level of FUS.

**Figure 3:**
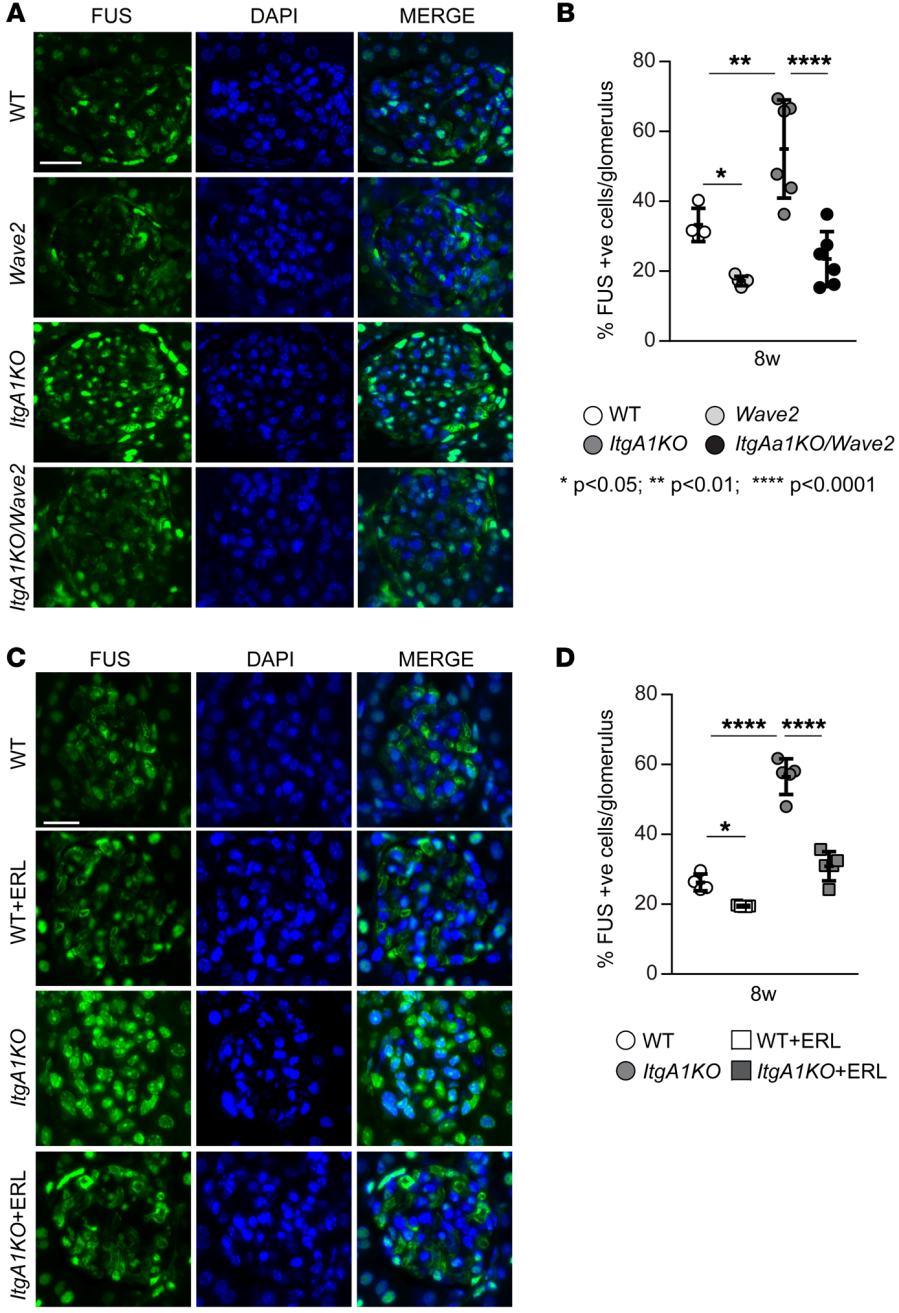
EGFR contributes to nuclear FUS translocation in ADR-induced injury. (**A** and **C**) Representative confocal images of kidneys from the mice indicated 8 weeks after ADR injections stained with anti-FUS antibody (green) or DAPI (blue). Scale bars: 15 μm. (**B** and **D**) The number of FUS-positive glomerular cells was counted and expressed as percentage of FUS-positive cells per glomerulus. Values are the mean ± SD, and symbols represent individual kidneys (**B**, *n* = 4 WT, *n* = 6 *ItgA1KO*, *n* = 5 *Wave2*, *n* = 6 *ItgA1KO/Wave2*; **D**, *n* = 4 WT, *n* = 5 *ItgA1KO*, *n* = 5 WT+ERL, *n* = 5 *ItgA1KO*+ERL, with an average of at least 4 glomeruli per kidney). Statistical analysis: 1-way ANOVA followed by Dunnett’s multiple-comparison test (**B** and **D**).

To determine whether EGFR kinase activity positively regulates nuclear translocation of FUS in injured kidneys, we evaluated the levels of phosphorylated EGFR in injured glomeruli by immunofluorescence. Significantly higher levels of phosphorylated EGFR were detected in the glomeruli of ADR-injured *ItgA1KO* mice compared with injured WT mice (Supplemental Figure 1, A and B; supplemental material available online with this article; https://doi.org/10.1172/JCI175158DS1). *ItgA1KO* mice crossed onto the *Wave2* background or treated with erlotinib showed significant reduction of activated EGFR upon injury (Supplemental Figure 1, A and B). Consistent with the levels of activated EGFR, analysis of kidneys 8 weeks after ADR injury revealed significantly higher levels of nuclear FUS in glomeruli of *ItgA1KO* mice, which were significantly decreased in *ItgA1KO/Wave2* mice or following erlotinib treatment (Figure 3, A–D). Thus, injury-mediated EGFR activation leads to increased FUS nuclear localization.

### FUSR521G mice show reduced ADR-induced glomerular injury.

To better define the role of nuclear FUS in regulating fibrotic responses, we induced glomerular injury in mice that express human FUS mutated in the NLS (R521G), which prevents its nuclear translocation (18). This mouse carries a CAG promoter, a floxed LacZ gene, the human FUS cDNA with the R521G mutation, and an IRES-EGFP (enhanced green fluorescent protein) coding sequence (18). We crossed this mouse with the *Pdgfrb-cre* mouse (19) to generate *cagFUSR521G; Pdgfrb-cre* mice (hereafter referred to as *FUSR521G*) (Supplemental Figure 2A), which express PGDFR-β in glomeruli (including mesangial cells [ref. 20]). Staining of kidneys from control (*Cre*) and *FUSR521G* mice (Supplemental Figure 2B) or Western blot analysis of kidney lysates (Supplemental Figure 2C) with anti-GFP antibody showed positive GFP staining or bands in glomeruli or kidney cortices of *FUSR521G* mice only. To ensure that expression of mutated FUS did not affect the basal levels of activated EGFR, we stained kidney sections with anti–phosphorylated EGFR. No overall differences in the basal levels of phosphorylated EGFR were observed in glomeruli of *Cre* or *FUSR521G* mice (Supplemental Figure 2, D and E).

**Figure 4:**
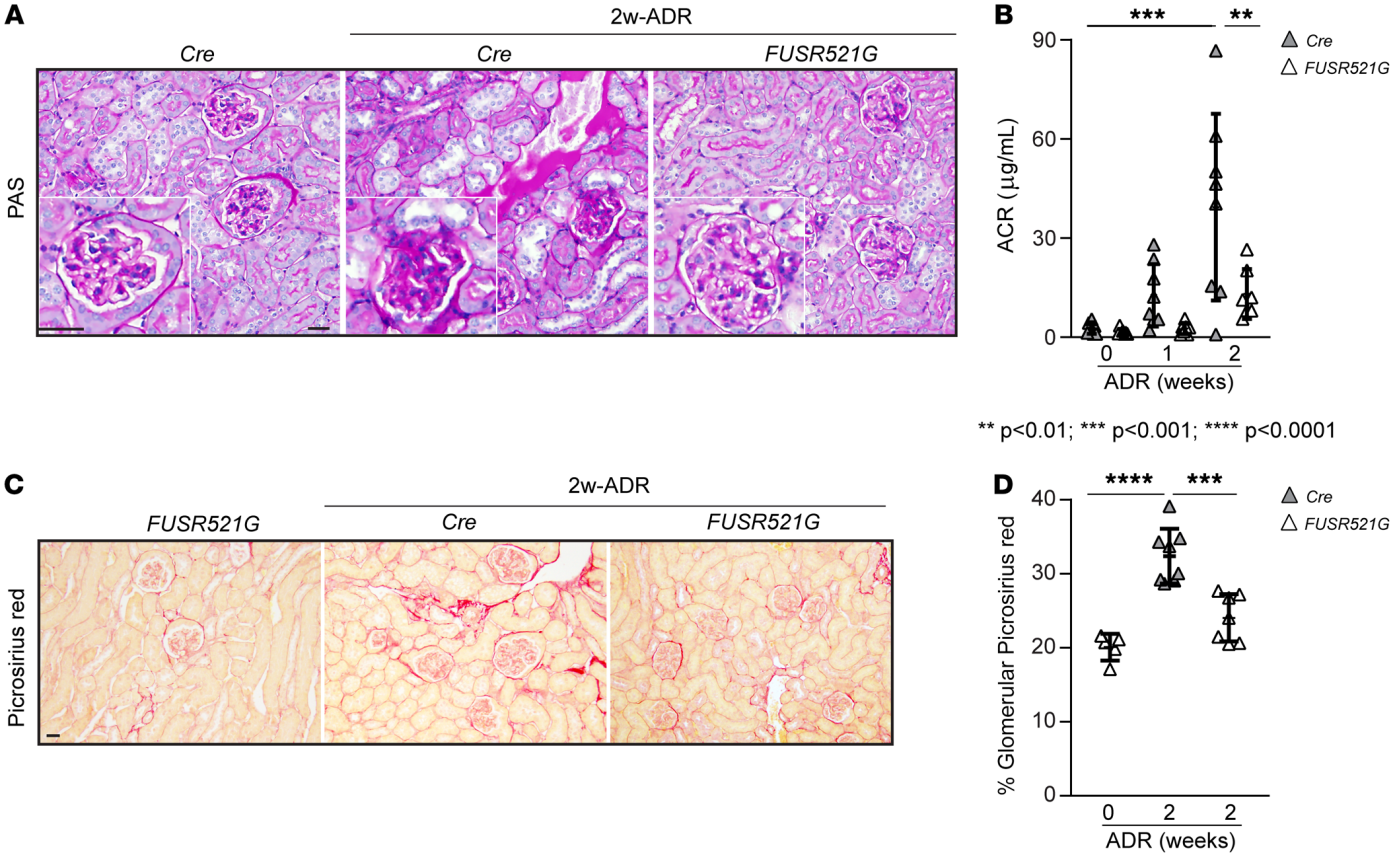
Reduced ADR-induced glomerulosclerosis in *FUSR521G* mice. (**A** and **C**) Representative images of periodic acid–Schiff–stained (**A**) or Picrosirius red–stained (**C**) kidney sections from uninjured (*Cre* or *FUSR521G*) mice and control (*Cre*) or *FUSR521G* mice treated with ADR for 2 weeks. Scale bars: 25 μm. (**B**) ACR was evaluated at 0, 1, and 2 weeks after ADR injection. Values are the mean ± SD, and symbols represent individual mice (*n* = 8 *Cre*, *n* = 7 *FUSR521G*). (**D**) The amount of glomerular fibrillar collagen was evaluated using ImageJ as described in Methods. Values are the mean ± SD, and symbols represent individual kidneys (*n* = 5 *Cre* 0-wk, *n* = 8 *Cre* 2-wk, *n* = 7 *FUSR521G*, with an average of at least 12 glomeruli per kidney). Statistical analysis: 1-way ANOVA followed by Dunnett’s multiple-comparison test (**B** and **D**).

Next, we induced ADR-mediated glomerular injury in control and *FUSR521G* mice. As these mice are on the C57BL/6J background, we followed the protocol described by Heikkilä et al. (21) and sacrificed the mice 2 weeks after injection. Compared with control (*Cre*) mice, *FUSR521G* mice showed reduced glomerular injury and accumulation of proteinaceous casts and significantly decreased albumin/creatinine ratio following injury (Figure 4, A and B). This protective effect was also accompanied by overall decreased Picrosirius red staining (Figure 4, C and D).

### FUSR521G mice show decreased nuclear FUS levels and glomerulosclerosis following kidney injury.

**Figure 5:**
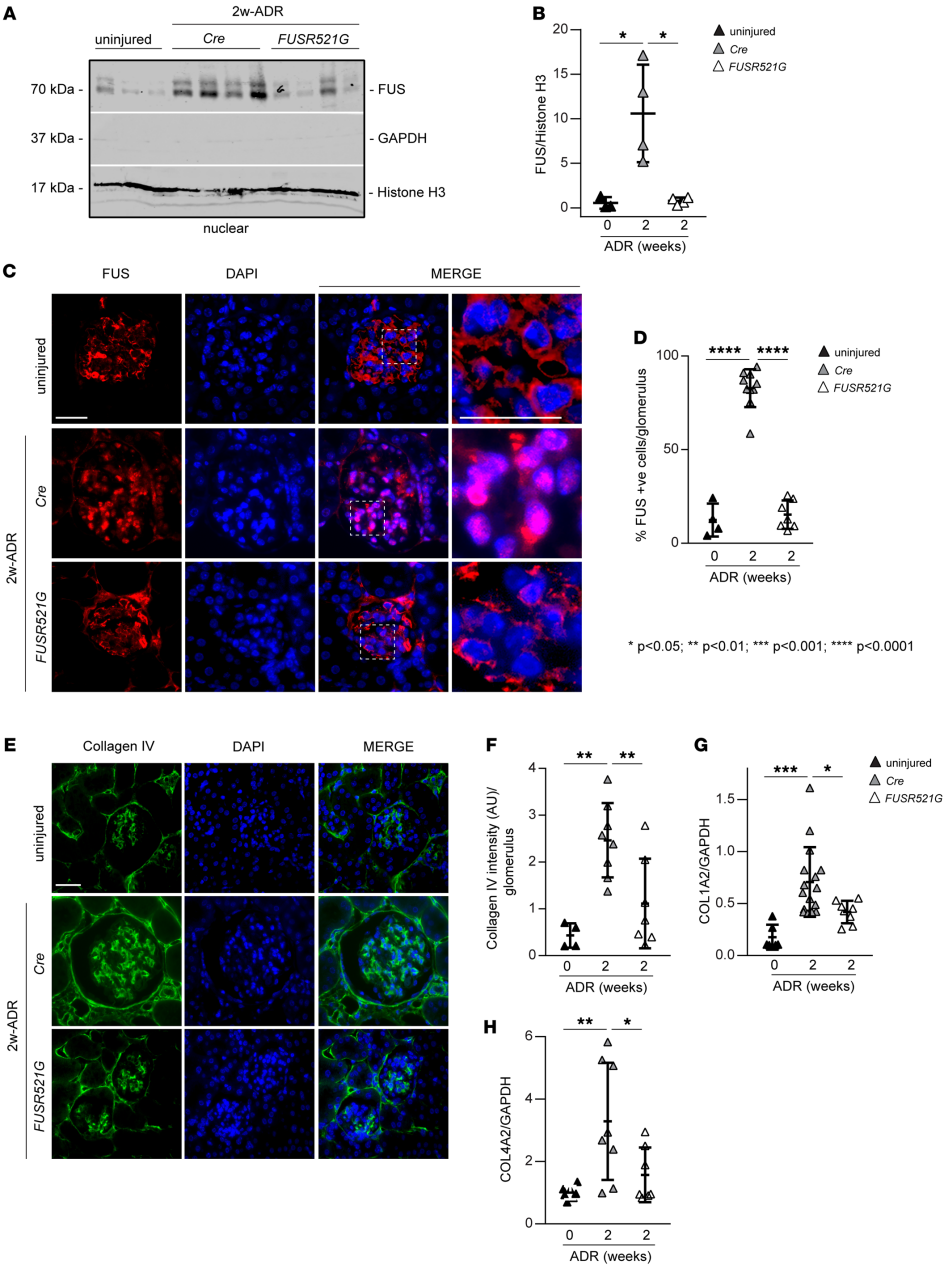
Reduced ADR-induced FUS nuclear translocation in *FUSR521G* mice. (**A**) Nuclear fractions (50 μg/lane) of kidney cortices from uninjured mice and mice treated with ADR for 2 weeks were analyzed by Western blot for levels of FUS. Histone H3 and GAPDH were used to verify the purity of nuclear and nonnuclear fractions, respectively. (**B**) FUS bands were quantified by densitometry analysis, and values were expressed as FUS/histone H3 ratio. Values are the mean ± SD, and symbols represent individual kidneys (*n* = 3 uninjured, *n* = 4 Cre, *n* = 4 *FUSR521G*). (**C**) Representative confocal images of kidneys from uninjured mice or mice treated with ADR for 2 weeks stained with anti-FUS antibody (red) or DAPI (blue). Scale bars: 20 μm. (**D**) The number of FUS-positive glomerular cells was counted and expressed as percentage of FUS-positive cells per glomerulus. Values are the mean ± SD, and symbols represent individual kidneys (*n* = 4 uninjured, *n* = 10 *Cre*, *n* = 7 *FUSR521G*, with an average of at least 10 glomeruli per kidney). (**E**) Representative images of kidney sections from uninjured mice or mice treated with ADR for 2 weeks stained with anti–collagen IV antibody. Scale bar: 20 μm. (**F**) The intensity of glomerular collagen IV was evaluated using ImageJ as described in Methods. Values are the mean ± SD, and symbols represent individual kidneys (*n* = 4 uninjured, *n* = 8 *Cre*, *n* = 7 *FUSR521G*, with an average of at least 10 glomeruli per kidney). (**G** and **H**) mRNA expression of *Col1A2* (*n* = 6 uninjured, *n* = 16 *Cre*, *n* = 8 *FUSR521G*) and *Col4A2* (*n* = 7 uninjured, *n* = 8 *Cre*, *n* = 7 *FUSR521G*) chains in kidney cortices of the mice indicated was analyzed by reverse transcription real-time quantitative PCR. Values are the mean ± SD, and symbols represent individual kidneys. Statistical analysis: 1-way ANOVA followed by Dunnett’s multiple-comparison test (**B**, **D**, and **F**–**H**).

To determine whether the protective effect observed in ADR-injured *FUSR521G* mice was due to reduced nuclear levels of FUS, we analyzed kidney nuclear fractions of uninjured as well as injured *Cre* control and *FUSR521G* mice by Western blot. Significantly higher levels of nuclear FUS were detected in ADR-treated *Cre* but not ADR-treated *FUSR521G* mice, compared with injured mice (Figure 5, A and B). Immunofluorescence analysis confirmed a significant increase in the number of FUS-positive nuclei in glomeruli of injured *Cre* mice compared with that detected in injured *FUSR521G* mice (Figure 5, C and D). Consistent with reduced FUS nuclear levels, *FUSR521G* mice also showed significant reduction in glomerular collagen IV deposition (Figure 5, E and F) and mRNA levels of *Col1A2* and *Col4A2* (Figure 5, G and H) compared with injured *Cre* mice.

### Pharmacologic inhibition of FUS nuclear translocation ameliorates ADR-mediated glomerular injury.

To translate the genetic findings to a more clinically relevant setting, we used a cell-penetrating peptide inhibitor of FUS nuclear translocation that we recently generated (11). This penetrating chimeric peptide (named CP-FUS-NLS), but not the mutated inactive peptide (CP-mutFUS-NLS), inhibits FUS nuclear translocation in cells by preventing its interaction with transportin 1/karyopherin β2. As we showed that CP-FUS-NLS prevents EGF-induced FUS nuclear translocation and collagen production by mesangial cells in vitro (11), we tested whether this peptide exerts an antifibrotic action in vivo.

First, we determined whether this peptide reaches the kidneys by performing an acute injection of CP-FUS-NSL conjugated to fluorescein amidite. To do this, the fluorescent peptide was injected every 2 hours i.p. (1 mg/kg BW) for a total of 6 hours. Two hours after the last injection, the kidneys were collected, and frozen kidney sections were analyzed under an epifluorescence microscope. Compared with vehicle-treated mice, kidneys from mice treated with fluorescein amidite–conjugated CP-FUS-NSL showed green fluorescence in both tubules and glomeruli (Supplemental Figure 3, A and B), indicating that this peptide reaches the kidneys.

Next, we induced ADR-mediated glomerular injury in WT male BALB/c mice. Mice were then divided into 3 groups: one group received vehicle (PBS), one group received CP-FUS-NSL (1 mg/kg — which corresponds to 0.3 nM — via i.p. injection twice a day, 3 times per week), and one group received CP-mutFUS-NSL (0.95 mg/kg — which corresponds to 0.3 nM — via i.p. injection twice a day, 3 times per week). After 2 weeks the mice were sacrificed and organs collected for analysis.

**Figure 6:**
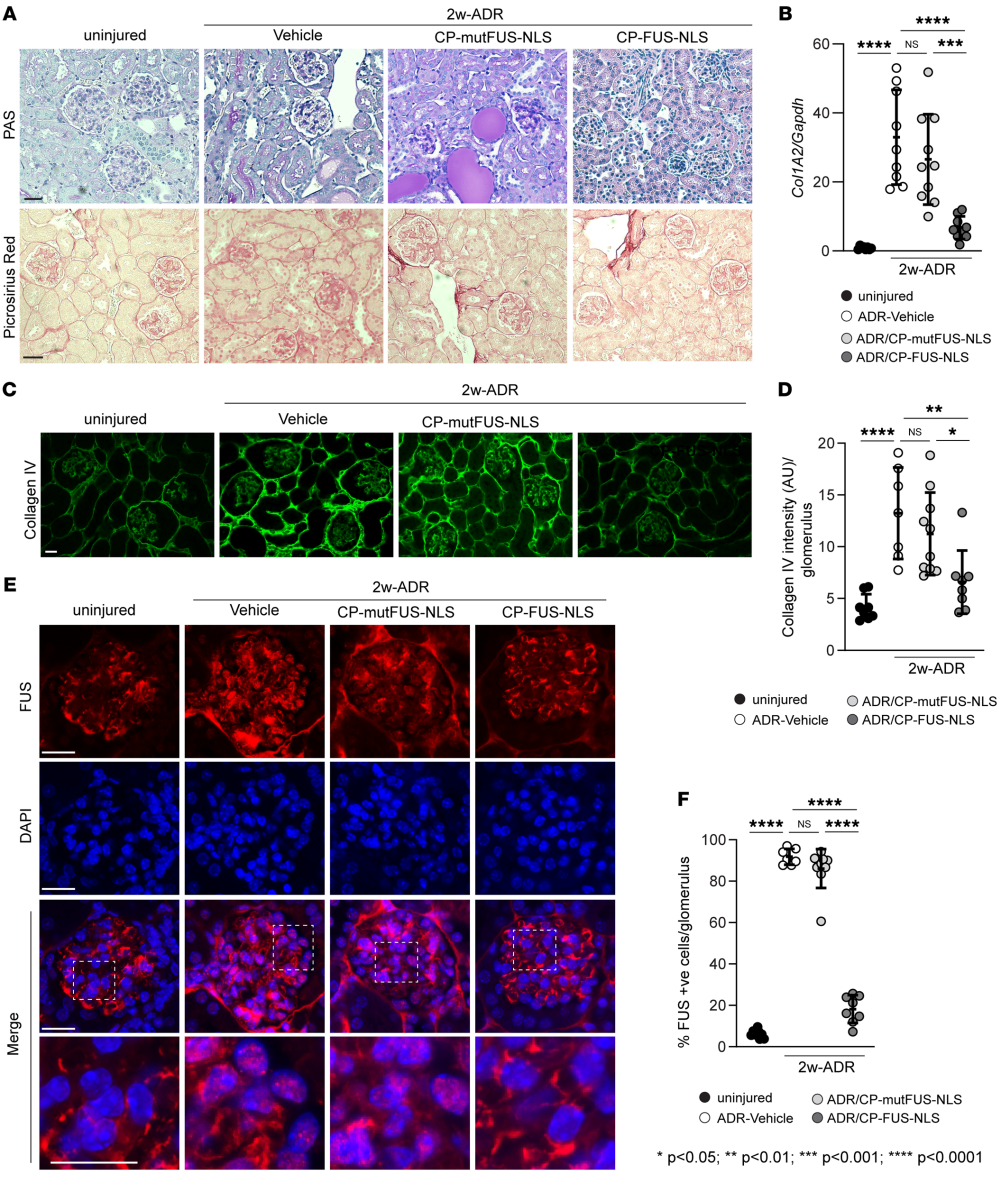
Pharmacologic inhibition of FUS nuclear translocation ameliorates ADR-induced glomerulosclerosis. (**A**) Representative images of periodic acid–Schiff–stained or Picrosirius red–stained kidney sections from uninjured mice and mice treated for 2 weeks with ADR alone or in combination with CP-mut-FUS-NLS or CP-FUS-NLS. Scale bars: 25 μm. (**B**) mRNA expression of *Col1A2* chain in kidney cortices of the mice indicated was analyzed by reverse transcription quantitative PCR. Values are the mean ± SD, and symbols represent individual kidneys (*n* = 8 uninjured, *n* = 9 ADR, *n* = 10 ADR+CP-mutFUS-NLS, *n* = 9 ADR+CP-FUS-NLS). (**C**) Representative images of kidney sections from the mice described in **A** stained with anti–collagen IV antibody. Scale bar: 20 μm. (**D**) The intensity of glomerular collagen IV was evaluated using ImageJ as described in Methods. Values are the mean ± SD, and symbols represent individual kidneys (*n* = 8 uninjured, *n* = 7 ADR, *n* = 10 ADR+CP-mutFUS-NLS, *n* = 8 ADR+CP-FUS-NLS, with an average of at least 10 glomeruli per kidney). (**E**) Representative images of kidneys from the mice described in **A** stained with anti-FUS antibody (green) or DAPI (blue). Scale bars: 20 μm. (**F**) The number of FUS-positive glomerular cells was counted and expressed as percentage of FUS-positive cells per glomerulus. Values are the mean ± SD, and symbols represent individual kidneys (*n* = 8 uninjured, *n* = 7 ADR, *n* = 10 ADR+CP-mutFUS-NLS, *n* = 8 ADR+CP-FUS-NLS, with an average of at least 10 glomeruli per kidney). Statistical analysis: 1-way ANOVA followed by Dunnett’s multiple-comparison test (**B**, **D**, and **F**).

Mice with ADR injury treated with CP-FUS-NLS showed reduced glomerular injury (Figure 6A) compared with injured mice treated with vehicle or CP-mutFUS-NLS, indicating that CP-FUS-NLS decreased glomerular injury.

Picrosirius red staining and real-time quantitative PCR analysis showed increased fibrillar collagen deposition and *Col1A2* mRNA in injured mice treated with vehicle or CP-mutFUS-NLS compared with uninjured mice (Figure 6, A and B). In contrast, significant reduction of fibrillar collagen deposition/synthesis was detected in injured mice treated with CP-FUS-NLS (Figure 6, A and B). Similar results were obtained when kidney slides were stained with anti–collagen IV antibody (Figure 6, C and D), supporting that CP-FUS-NLS plays an antifibrotic action in vivo.

To determine whether the protective role of CP-FUS-NLS resides in its ability to prevent FUS nuclear translocation in vivo, we stained frozen kidney sections with anti-FUS antibody. Compared with uninjured mice, we detected a significant increase in the number of FUS-positive cells in glomeruli of injured mice treated with vehicle or CP-FUS-NLS (Figure 6, E and F). Importantly, the number of FUS-positive glomerular cells in injured mice treated with CP-FUS-NLS was similar to that detected in injured mice (Figure 6, E and F).

### FUS nuclear translocation is upregulated in liver fibrosis.

**Figure 7:**
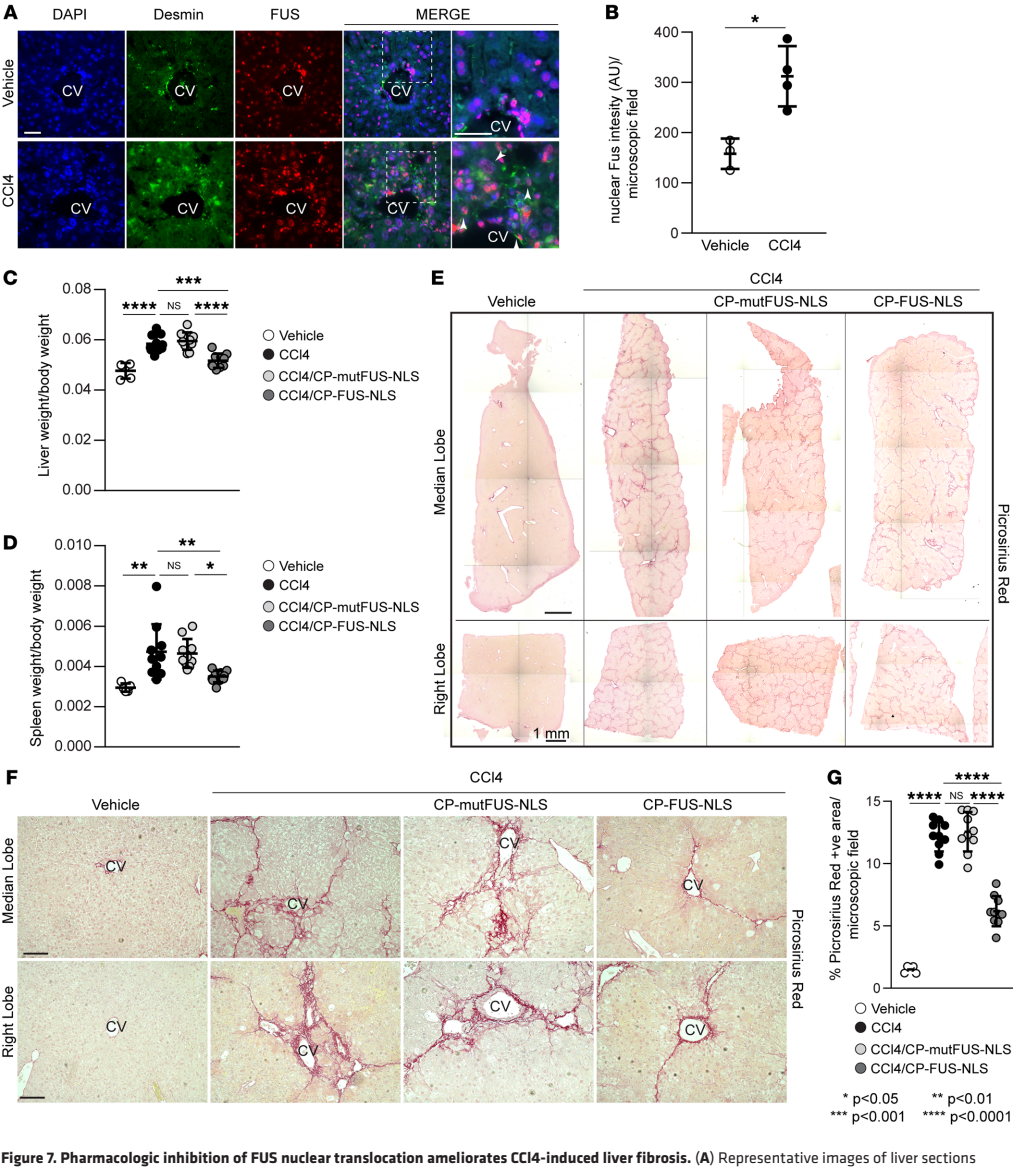
Pharmacologic inhibition of FUS nuclear translocation ameliorates CCl4-induced liver fibrosis. (**A**) Representative images of liver sections from WT mice treated with CCl4 for 6 weeks stained for desmin (green), FUS (red), and DAPI (blue). CV, central vein. Arrowheads indicate desmin-positive cells. Scale bars: 20 μm. (**B**) Nuclear FUS intensity per microscopic field was calculated using ImageJ. Values are the mean ± SD, and symbols represent individual livers (*n* = 3 vehicle, *n* = 4 CCl4, with an average of at least 120 cells per microscopic field per liver). (**C** and **D**) Liver/body weight and spleen/body weight ratios in uninjured (vehicle) mice and mice treated for 6 weeks with CCl4 alone or in combination with CP-mut-FUS-NLS or CP-FUS-NLS. Values are the mean ± SD, and symbols represent individual livers or spleens (*n* = 5 vehicle, *n* = 10 CCl4, *n* = 10 CCl4+CP-mutFUS-NLS, *n* = 10 CCl4+CP-FUS-NLS). (**E**) Reconstruction of histologic images of median and right lobes of livers from the mice described in **C** stained with Picrosirius red staining. Scale bars: 1 mm. (**F**) Representative images of Picrosirius red–stained liver median and right lobes. Scale bars: 30 μm. (**G**) The amount of fibrillar collagen was evaluated using ImageJ software as described in Methods. Values are the mean ± SD, and symbols represent individual livers (*n* = 5 vehicle, *n* = 10 CCl4, *n* = 10 CCl4+CP-mutFUS-NLS, *n* = 10 CCl4+CP-FUS-NLS, with an average of at least 5 microscopic fields per lobe). Statistical analysis: unpaired 2-tailed *t* test (**B**); 1-way ANOVA followed by Dunnett’s multiple-comparison test (**C**, **D**, and **G**).

To determine whether FUS is an important driver of fibrosis in other organs, we examined whether CP-FUS-NLS is beneficial in the setting of liver fibrosis. We focused on the liver for the following reasons: (a) EGFR activation plays a detrimental role in the development of liver fibrosis (22). (b) Analysis of livers of mice treated with CCl4, a well-established inducer of liver fibrosis (23), showed a significant increase in the number of FUS-positive cells compared with vehicle-treated cells (Figure 7, A and B); FUS-positive cells also stained for desmin (Figure 7A), a marker of hepatic stellate cells (HSCs) (24), which play a key role in the fibrosis process (25). (c) Fluorescent cells were detected in the livers of mice treated with fluorescein amidite–conjugated CP-FUS-NSL (Supplemental Figure 3C), indicating that this peptide reaches the liver.

### Pharmacologic inhibition of FUS nuclear translocation ameliorates CCl4-mediated liver fibrosis.

To induce liver fibrosis in mice, we used the well-established CCl4-induced liver fibrosis model. This is a simple, highly reproducible, and quick (fibrosis is evident within 4–12 weeks) model that leads to fibrosis in rodents with features that resemble those of human liver fibrosis (26). C57BL/6J WT male mice received i.p. injection of CCl4 or vehicle as control (corn oil) 3 times per week for a total of 6 weeks. After 3 weeks of treatment, CCl4-treated mice were divided into 3 groups: one group treated with PBS, one group treated with CP-mutFUS-NLS, and one group treated with CP-FUS-NLS via i.p. injection 3 times per week. After 6 weeks of CCl4 treatment, mice were sacrificed for organ collection. Following CCl4 exposure, mice treated with CCl4 alone or CP-mutFUS-NLS exhibited a significant increase in both liver/body weight and spleen/body weight ratios compared with corn oil–treated mice (Figure 7, A and B). In contrast, mice treated with CP-FUS-NLS showed liver/body weight and spleen/body weight ratios like those of corn oil–treated mice (Figure 7, C and D), suggesting that CP-FUS-NLS decreases CCl4-induced liver injury.

Analysis of liver sections stained with Picrosirius red staining showed a significant increase in the levels of fibrillar collagens in mice treated with CCl4 alone or CCl4/CP-mutFUS-NLS compared with corn oil–treated mice (Figure 7, E–G). CCl4-induced fibrosis was significantly attenuated by treatment of mice with CP-FUS-NLS, showing that CP-FUS-NLS exerts antifibrotic action in a mouse model of liver fibrosis.

### Pharmacologic inhibition of FUS nuclear translocation ameliorates CCl4-induced FUS nuclear translocation.

**Figure 8:**
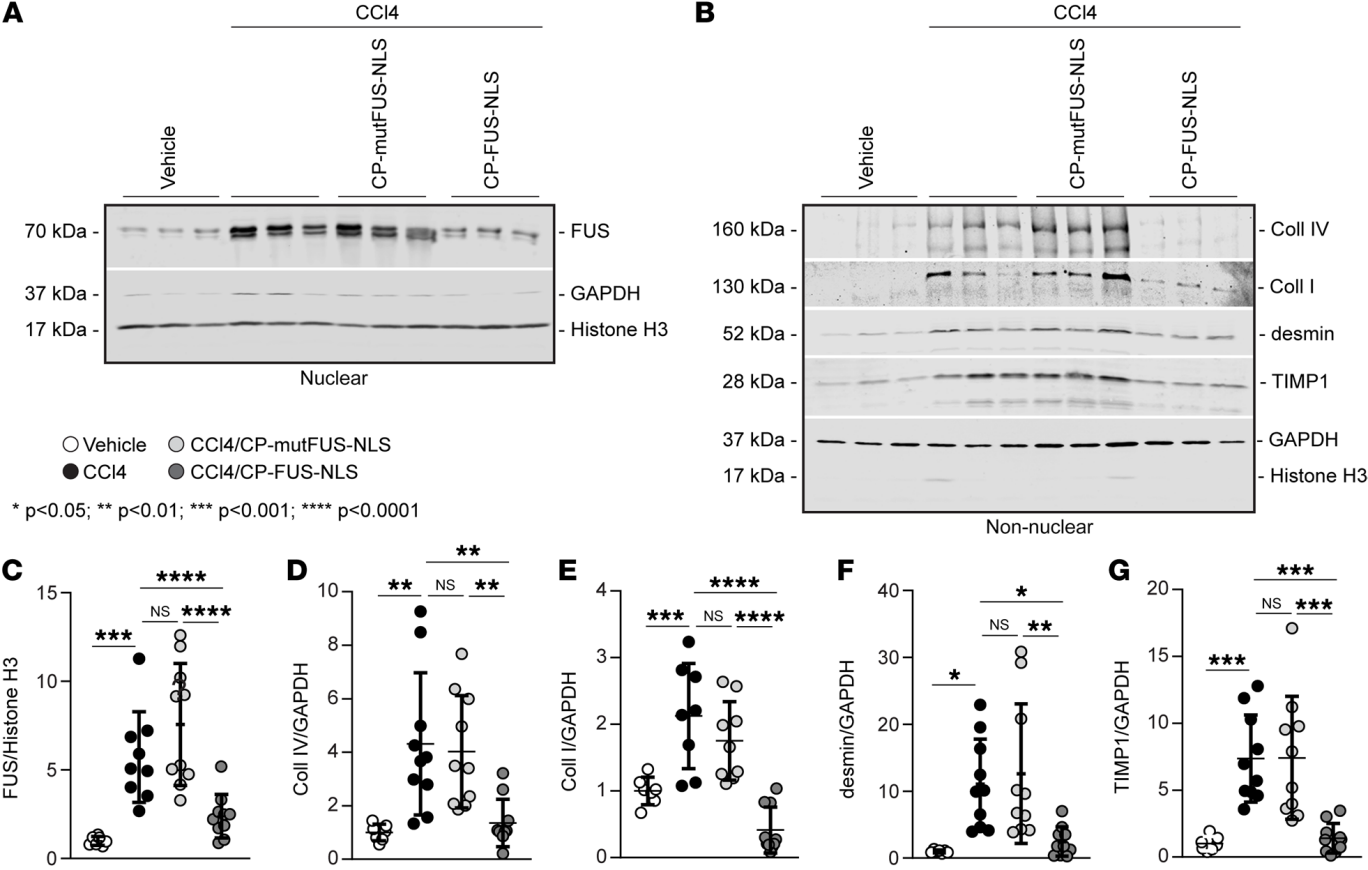
FUS nuclear translocation regulates the expression of fibrotic markers in CCl4-induced liver fibrosis. (**A**) Nuclear fractions (50 μg/lane) of livers from uninjured (vehicle) mice and mice treated for 6 weeks with CCl4 alone or in combination with CP-mutFUS-NLS or CP-FUS-NLS peptide were analyzed by Western blot for FUS levels. Histone H3 and GAPDH were used to verify the purity of nuclear fractions. (**B**) Nonnuclear fractions (50 μg/lane) of livers from the mice described in **A** were analyzed by Western blot for levels of collagens I and IV, desmin, and TIMP1. Histone H3 and GAPDH were used to verify the purity of nonnuclear fractions. (**C**–**G**) Immunoreactive bands were quantified by densitometric analysis, and values were expressed as ratios relative to histone H3 for nuclear proteins or GAPDH for nonnuclear proteins. Values are the mean ± SD, and symbols represent individual livers (*n* = 7 vehicle, *n* = 8–10 CCl4, *n* = 9–10 CCl4+CP-mutFUS-NLS, *n* = 10 CCl4+CP-FUS-NLS). Statistical analysis: 1-way ANOVA followed by Dunnett’s multiple-comparison test (**C**–**G**).

To determine whether CP-FUS-NLS prevents CCl4-induced FUS nuclear translocation, we investigated the levels of FUS in liver nuclear fractions of CCl4-treated mice by Western blot. Compared with corn oil–treated mice, significantly increased levels of nuclear FUS were detected in mice treated with CCl4 alone as well as those treated with CCl4/CP-mutFUS-NLS (Figure 8, A and C). By contrast, treatment of mice with CP-FUS-NLS resulted in a significant reduction in hepatic FUS nuclear levels (Figure 8, A and C). Consistent with this finding, Western blot analysis revealed significant reduction in the levels of hepatic collagen I and collagen IV levels in CP-FUS-NLS–treated mice compared with CCl4 alone– or CCl4/CP-mutFUS-NLS–treated mice (Figure 8, B, D, and E). Upon injury, HSCs activate and undergo cell proliferation. Reduced levels of desmin, a marker of HSCs, and tissue inhibitor of metalloproteinase-1 (TIMP1; a profibrotic marker upregulated in hepatic fibrogenesis) were also observed in CP-FUS-NLS–treated mice (Figure 8, B, F, and G), indicating decreased liver injury and HSC expansion.

### Pharmacologic inhibition of FUS nuclear translocation decreases fibrinogenic activity in murine HSC line JS1.

**Figure 9:**
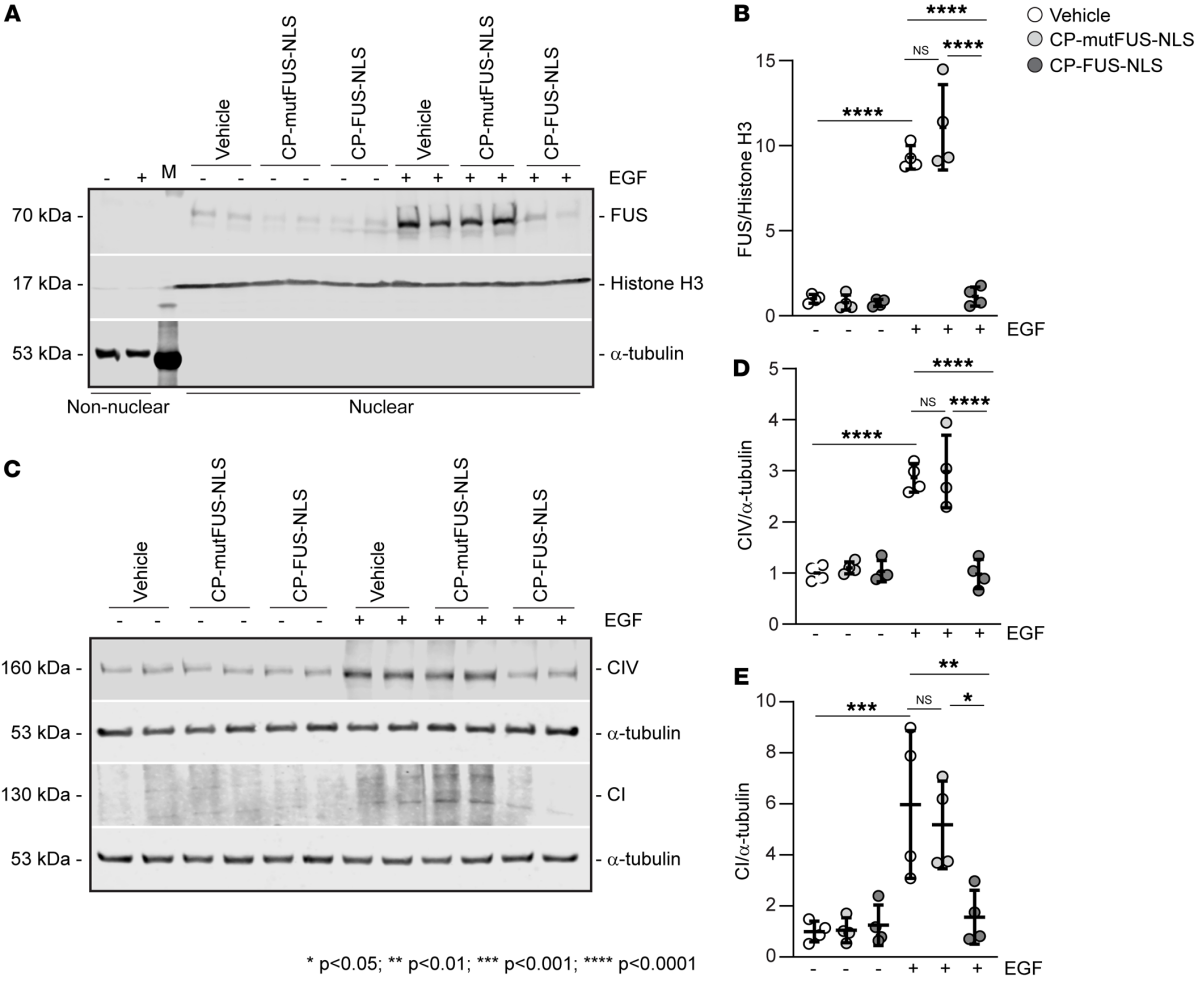
EGF promotes FUS nuclear translocation and fibrotic responses in HSCs. (**A**) Nuclear and nonnuclear fractions (50 μg/lane) of HSCs treated with vehicle or EGF alone or in combination with CP-mutFUS-NLS or CP-FUS-NLS peptides were analyzed by Western blot for FUS levels. Histone H3 and α-tubulin were used to verify the purity of nuclear and nonnuclear fractions, respectively. (**B**) FUS nuclear bands were quantified by densitometric analysis, and values were expressed as FUS/histone H3 ratio. Values are the mean ± SD, and symbols represent individual treatments (*n* = 4 for all treatments). Two experiments were performed in duplicate. (**C**) Total cell lysates (50 μg/lane) of the cells described in **A** were analyzed by Western blot for levels of collagens I and IV. α-Tubulin was used as loading control. (**D** and **E**) Collagen bands were quantified by densitometric analysis, and values were expressed as collagen I/ or collagen IV/α-tubulin ratio. Values are the mean ± SD, and symbols represent individual treatments (*n* = 4 for all treatments). Two experiments were performed in duplicate. Statistical analysis: 1-way ANOVA followed by Dunnett’s multiple-comparison test (**B**, **D**, and **E**).

Our in vivo data indicate that HSCs are a major cell type showing increased nuclear levels of FUS after liver injury (Supplemental Figure 4, A and B). This finding together with the observation that EGFR contributes to liver fibrosis (22, 27) and is upregulated in activated HSCs (28) prompted us to analyze the role of the EGFR/FUS axis in regulating fibrotic responses in HSCs. Treatment of the murine HSC line JS1 (29) with EGF stimulated EGFR activation in a time-dependent manner (Supplemental Figure 4, A and B). Treatment of JS1 cells with EGF also increased the levels of nuclear FUS, which were significantly reduced after treatment with CP-FUS-NLS, but not CP-mutFUS-NLS (Figure 9, A and B). Treatment with EGF also stimulated the production of collagens I and IV by JS1 cells, which was significantly dampened by treatment with CP-FUS-NLS (Figure 9, C–E). Thus, EGF-mediated fibrotic signaling in HSCs requires FUS nuclear translocation.

### FUS expression in individuals with kidney and liver fibrosis.

**Figure 10:**
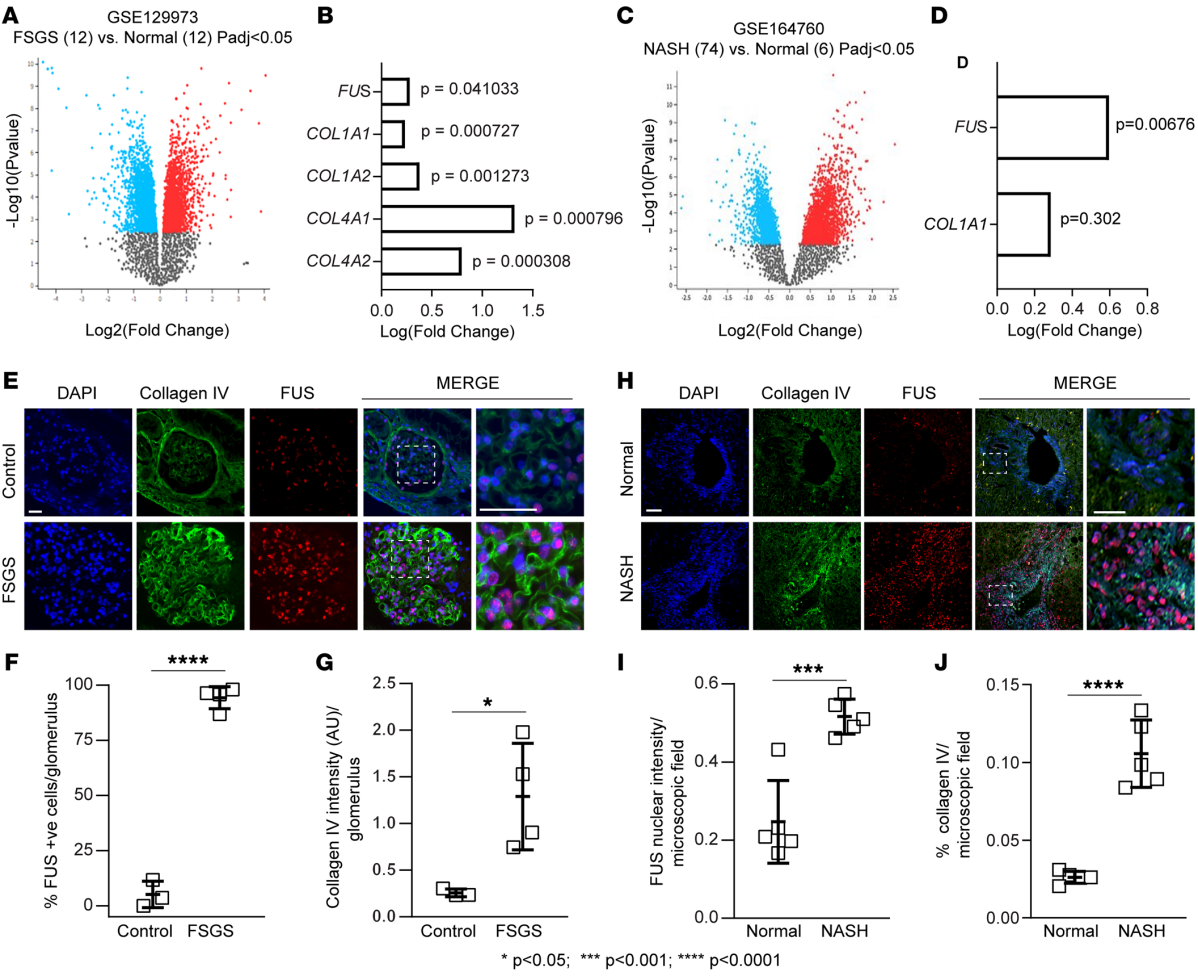
FUS mRNA is upregulated in kidneys and livers of individuals with kidney and liver disease. (**A**) Volcano plot of differentially expressed mRNAs in 12 kidney biopsies of individuals with FSGS versus 12 healthy controls (Normal). Gray dots represent genes with no significant differences; blue dots represent downregulated genes, and red dots upregulated genes, with fold change >1.0 and *P* value <0.05. (**B**) Examples of fibrotic genes upregulated in individuals with FSGS with fold change >1.0 and *P* value <0.05. (**C**) Volcano plot of differentially expressed mRNAs in 74 liver biopsies of individuals with NASH versus 6 healthy controls (Normal). Gray, blue, and red dots represent gene changes as described in **A**. (**D**) Examples of fibrotic genes upregulated in individuals with NASH with fold change >1.0 and *P* value <0.05 (for FUS only). (**E** and **H**) Representative images of kidney and liver tissue samples from controls or individuals with FSGS or NASH costained with anti-FUS and anti–collagen IV antibodies. Scale bars: 25 μm. (**F**) The number of nuclear FUS-positive glomerular cells was calculated, and values are expressed as percentage FUS-positive cells per glomerulus. Values are the mean ± SD, and symbols represent individual kidneys (*n* = 3 normal, *n* = 4 FSGS, with 2–6 glomeruli analyzed per biopsy). (**G**) Collagen IV intensity per glomerulus was evaluated and expressed as described in Methods. Values are the mean ± SD, and symbols represent individual kidneys (*n* = 3 normal, *n* = 4 FSGS, with 2–6 glomeruli analyzed per biopsy). (**I** and **J**) Nuclear FUS intensity (**I**) or collagen IV intensity (**J**) per microscopic field was evaluated and expressed as described in Methods. Values are the mean ± SD, and symbols represent individual livers (*n* = 5 normal, *n* = 5 NASH, with an average of 3 microscopic fields analyzed per biopsy). Statistical analysis: unpaired 2-tailed *t* test (**F**, **G**, **I**, and **J**).

To further validate our in vitro and in vivo findings, we evaluated a possible correlation between FUS and fibrosis markers in patients with focal segmental glomerulosclerosis (FSGS) or nonalcoholic steatohepatitis (NASH). We analyzed the differential gene expression in publicly available data sets (NCBI Gene Expression Omnibus [GEO] GSE129973, https://www.ncbi.nlm.nih.gov/geo/query/acc.cgi?acc=GSE129973) by comparing 12 kidney biopsies from male individuals with FSGS versus 12 healthy male controls (Figure 10A). Volcano plot analysis revealed several differentially regulated mRNAs between the 2 groups. Gene expression levels of *FUS*, *COL1A1*, *COL1A2*, *COL4A1*, and *COL4A2* were significantly upregulated in diseased kidneys compared with controls (Figure 10B). Next, we analyzed the differential expression of mRNAs from the GSE164760 database (https://www.ncbi.nlm.nih.gov/geo/query/acc.cgi?acc=GSE164760) by comparing 74 liver biopsies from individuals with NASH versus 6 healthy controls (Figure 10C). Among the differentially regulated mRNAs, *FUS* was significantly upregulated in diseased livers (Figure 10D), and *COL1A1* mRNA levels were higher, although not significantly so, in individuals with NASH (Figure 10D).

To analyze the levels of nuclear FUS and collagen in diseased organs, we costained livers or kidney sections from individuals with FSGS or NASH with anti-FUS and anti–collagen IV antibodies. Compared with control tissues, collagen IV staining was significantly increased in fibrotic kidneys and livers, which correlated with significantly increased nuclear levels of FUS in both injured tissues (Figure 10, E–J). In individuals with FSGS, FUS nuclear localization was detected primarily in sclerosed glomeruli (Figure 10E), while in individuals with NASH, positive staining was at portal triads near bile ducts, consistent with ductular reaction (Figure 10H). Thus, *FUS* mRNA and nuclear localization levels positively correlate with fibrotic gene mRNAs and organ injury in individuals with fibrotic diseases.

## Discussion

The goal of this study was to determine the role of FUS in promoting fibrotic responses in vivo and determine the beneficial effects of preventing FUS nuclear translocation in two different models of organ fibrosis. Herein, we provide genetic and pharmacologic evidence that preventing FUS nuclear translocation ameliorates fibrosis in murine models of chemical-induced kidney and liver injury.

FUS is a nuclear DNA/RNA-binding protein that regulates different steps of gene expression, including transcription, splicing, and mRNA transport (30). Accumulation of cytoplasmic inclusions containing FUS is a common hallmark of frontotemporal lobar degeneration and amyotrophic lateral sclerosis (ALS) neuropathology. Cytoplasmic retention of FUS in neurons and glial cells of the spinal cord leads to formation of stress granules as well as reduced translation of genes associated with mitochondrial function and overall neurotoxicity (31). The nuclear translocation of FUS is mediated by interaction of the NLS in FUS with transportin 1/karyopherin β2. Mutations of FUS within the NLS that prevent its interaction with transportin lead to cytoplasmic retention of FUS and have been linked to familial ALS-FUS (32). In addition to mutations, posttranslational modifications of FUS can regulate its nuclear translocation and function. To this end, the C-terminal tyrosine residue at position 526 of FUS is crucial for normal nuclear import. Src-mediated phosphorylation of this residue reduces interactions with transportin 1, thus reducing FUS nuclear translocation and potentially contributing to the development of frontotemporal lobular degeneration (33).

In contrast to these findings, we showed that in kidney cells, EGFR-induced phosphorylation of FUS on tyrosines 6 and 296 promotes FUS nuclear translocation (11). Phosphorylated FUS binds to the collagen IV promoter, commencing its gene transcription. Consistent with a positive role of FUS tyrosine phosphorylation in promoting its nuclear translocation, we showed that treatment of kidney cells with erlotinib or cells carrying mutated tyrosines 6 and 296 had reduced EGFR-mediated FUS nuclear translocation and overall decreased collagen production (11). Thus, preventing FUS nuclear translocation can result in both beneficial and detrimental effects that are cell and organ specific.

In agreement with our in vitro finding that EGFR induces fibrosis by promoting FUS nuclear translocation and transcription of fibrotic genes, we provide in vivo genetic (*Wave2*) and pharmacologic (erlotinib) evidence that inhibition of EGFR kinase activity reduces FUS nuclear translocation and overall development of fibrosis following kidney injury. Our results agree with published data that EGFR activation contributes to kidney fibrosis and reveal that FUS is a key downstream mediator of EGFR-induced kidney fibrosis. This finding is clinically relevant as inhibition of EGFR with small-molecule kinase inhibitors in humans leads to severe side effects. These are especially severe in epithelial tissues, including the skin and hair follicle, where EGFR is highly expressed (34). Moreover, although rarely observed, erlotinib can cause interstitial lung disease (35). Thus, targeting downstream pathways directly regulated by EGFR might offer better-tolerated and safer antifibrotic therapy.

We provide evidence that mice carrying a mutated FUS unable to translocate to the nucleus (*FUSR521G* mice) develop less fibrosis following ADR-induced kidney injury compared with WT mice. This finding, together with the observation that EGFR is activated/phosphorylated in injured *FUSR521G* mice, further indicates that FUS is a key downstream mediator of EGFR-induced kidney fibrosis. To further corroborate this genetic mouse model, we reveal that treatment of mice with the recently developed CP-FUS-NLS peptide (11) ameliorates both liver and kidney fibrosis by downregulating FUS nuclear translocation in glomeruli and HSCs and, in turn, the synthesis and transcription of fibrotic genes. In addition to promoting ECM synthesis, FUS could contribute to organ injury by regulating inflammatory responses. In astrocytes, overexpression of WT FUS leads to enhanced expression of proinflammatory genes (36). Although this study did not examine whether nuclear translocation of FUS is required for its proinflammatory action, the finding that reactive proinflammatory astrocytes upregulate EGFR expression (37) suggests that an EGFR/FUS axis could regulate inflammatory responses in these cells.

Our finding that preventing FUS nuclear translocation has a beneficial antifibrotic effect contrasts with the finding that downregulation of FUS by siRNA in mouse skin epithelial cells or overexpression of FUSR521G in motor neuron–like cells leads to decreased transcription and activity of manganese superoxide dismutase (38), thus enhancing the production of profibrotic reactive oxygen species. A possible explanation for this discrepancy is that we induced expression of FUSR521G primarily in glomerular cells. Taken together, these data suggest that preventing FUS nuclear translocation has both deleterious and protective effects that are disease and cell type specific.

Although we focused on the role of FUS in glomerular injury (based on our finding that FUS is expressed by mesangial cells) (11), the finding that liver fibrosis is also mediated by nuclear FUS suggests that FUS plays a more global role in regulating fibrotic responses driven by EGFR activation. In the kidney, prolonged and/or aberrant EGFR signaling is a key determinant of progressive acute and chronic tubular injury (5, 39). *Dsk5*-mutant mice with a gain-of-function allele that increases basal EGFR kinase activity have increased spontaneous glomerular and tubulointerstitial injury and overall increased glomerular FUS nuclear levels (11). Thus, it is conceivable that FUS might contribute to both glomerular and tubular injury that progresses to fibrosis.

Our study suggests that systemic inhibition of FUS nuclear translocation might represent a therapeutic approach to target FUS localization in the setting of fibrosis. ADR- or CCl4-challenged mice tolerated treatment with CP-FUS-NLS up to 3 weeks without visible side effects. It is, however, still unclear whether this peptide can be used for a longer treatment regime, after injury and/or establishment of fibrosis, and whether it could induce severe side effects by affecting the central nervous system or skin fibroblast function. Cell-penetrating peptides homing to selective tissues, including vascular endothelium, dendritic cells, and cardiac myocytes, to name a few, have been generated (40, 41). Although identifying selective receptors that can facilitate kidney (e.g., mesangial cells) and liver (e.g., HSCs) uptake of cell-penetrating peptides might be challenging, our study clearly shows that cell-penetrating peptides able to prevent FUS/transportin interaction can be used in vivo to ameliorate organ injury and fibrosis. Thus, our study points to nuclear translocation of FUS as a targetable step in organ fibrosis.

## Methods

### Sex as a biological variable

All animal studies were conducted on sexually mature male mice, as they are more susceptible than female mice to drug-induced organ injury. Analysis of human samples included both sexes.

### Study design

The object of this paper was to investigate the role of FUS in promoting organ fibrosis as well as to determine whether preventing FUS nuclear translocation with cell-penetrating peptides prevented the development of organ fibrosis. Each biochemical experiment was performed at least twice with similar results**)**. Western blot, immunofluorescence, immunohistochemistry, and quantitative PCR were used to evaluate FUS cellular localization and FUS-activated fibrotic pathways. The investigators were blinded to the allocation of the groups for evaluation of mesangial sclerosis index, collagen deposition, and FUS nuclear localization (periodic acid–Schiff, Picrosirius red, and immunofluorescence staining). All other experiments were performed in a nonblinded manner. For in vivo studies, mice were randomly divided into different groups before treatments and were humanely sacrificed at defined study endpoints.

### Design and development of cell-penetrating nuclear import inhibitors of FUS

A cell-penetrating chimeric peptide, an inhibitor of nuclear translocation of FUS, and its cell-penetrating inactive control peptide were designed as described previously (11). The synthesis of a WT peptide, CP-FUS-NLS (AAVALLPAVLLALLAPSRGEHRQDRRERPY; 30 aa; MW 3,340 Da), and its inactive control peptide, CP-mutFUS-NLS (AAVALLPAVLLALLAPSEGEHREDREERGA; 30 aa; MW 3,155 Da), was conducted on a Focus XC automated peptide synthesizer (AAPPTec) using standard Fmoc chemistry protocols. The fluorescein amidite–labeled (FAM-labeled) CP-FUS-NLS peptide was obtained by coupling of 5(6)-carboxyfluorescein to the N-terminus of peptide using standard coupling protocol.

After chain assembly was completed, crude peptides were removed from resin with a TFA cleavage cocktail and purified by dialysis against double-distilled water in 2 kDa membrane (Spectra/Por 7, Spectrum Laboratories). Purity and structure of the final products were verified by analytical C18 RPHPLC (Beckman Coulter GOLD System) and MALDI mass spectroscopy (Voyager Elite, PerSeptive Biosystems).

To visualize CP-FUS-NLS tissue distribution, 6- to 8-week-old male BALB/c WT mice received FAM-labeled CP-FUS-NLS (1 mg/kg) or vehicle (PBS) via i.p. injections every 2 hours for a total of 6 hours. Two hours after the last injection, mice were sacrificed, and kidneys and livers were collected. Parts of tissues were immediately frozen, and some parts were embedded in OCT compound.

### Mouse injury models

#### Kidney injury.

Six- to eight-week-old male BALB/c WT, *ItgA1KO*, *Wave2*, and *ItgA1KO/Wave2* mice received a single i.v. injection of ADR (10 mg/kg; D1515, MilliporeSigma) and were sacrificed 8 weeks after injection. To generate BALB/c *ItgA1KO/Wave2*, *Wave2* mice, which have deficient EGFR kinase activity (42), were bred onto a BALB/c background for at least 10 generations and then crossed with BALB/c *ItgA1KO* mice (described in ref. 3). The *ItgA1het/Wave2het* mice were crossed among themselves to generate the 4 genotypes indicated above. Mice were sacrificed 8 weeks after ADR injection.

In some experiments, BALB/c male WT mice received a single i.v. injection of ADR (10 mg/kg) and were divided into 3 groups. One group received vehicle (PBS), one group received CP-FUS-NSL (1 mg/kg i.p., which corresponds to 0.3 nM), and one group received CP-mutFUS-NLS (0.95 mg/kg i.p., which corresponds to 0.3 nM). Mice received 2 i.p. injections per day 3 times a week for the entire duration of the experiment. Two weeks after ADR injection, kidneys were harvested and used for RNA and Western analysis or immunohistochemistry.

To generate mice that express human FUS carrying the R521G mutation in glomerular cells, C57BL/6J *CAG-Z-FUSR521G-IRES-EGFP* mice (strain 028021, The Jackson Laboratory) were crossed with *Pdgfrb-cre* mice [*Tg(Pdgfrb-Cre)35Vli*] (19) and backcrossed to C57BL/6J background for at least 10 generations to generate *cagFUSR521G Pdgfrb-cre* (referred to as *FUSR521G*) mice. Six- to eight-week-old male *FUSR521G* and control mice (e.g., C57BL/6J WT or *Pdgfrb-cre* mice) received a single i.v. injection of ADR (22–24 mg/kg) and 2 daily i.p. injections of 5% glucose-saline solution (0.5 mL) for 7 consecutive days (21). Mice were sacrificed 2 weeks after ADR injection.

#### Liver injury.

Six- to eight-week-old male C57BL/6J WT mice received an i.p. injection of corn oil (100 μL) or 10% CCl4 (289116, MilliporeSigma) diluted in corn oil, 3 times per week for 6 weeks. Some CCl4-treated mice received CP-mutFUS-NLS (1 mg/kg, which corresponds to 0.3 nM) or CP-FUS-NLS (0.95 mg/kg, which corresponds to 0.3 nM) twice a day, 3 times per week from week 3 to week 6 of CCl4 treatment. Mice were then sacrificed for organ collection.

### Clinical parameters and morphologic analysis

For the analysis of albuminuria, spot urine was collected before ADR injection and at time of sacrifice. Urine albumin and creatinine levels were measured using the ELISA Albuwell M test and kit (Exocel Inc.), and the albumin/creatinine ratio was expressed as micrograms per milligram.

For histologic analysis, kidneys or livers, removed immediately after sacrifice, were either fixed in 4% formaldehyde and embedded in paraffin for morphologic and immunohistochemistry analysis, or embedded in OCT compound for immunofluorescence or immediately frozen for Western blot analysis. The paraffin tissue sections were stained with periodic acid–Schiff, H&E, trichrome, and Picrosirius red for the evaluation of glomerular or hepatic injury. Mesangial sclerosis index was evaluated in a blinded fashion as previously described (43). Briefly, the percentage of mesangial matrix occupying each glomerulus was rated as 1 = 0%–24%, 2 = 25%–49%, 3 = 50%–74%, and 4 = >75%. Picrosirius red staining was quantified by evaluation of the percentage of red staining per microscopic field.

### Human samples

#### Human kidney biopsies.

Biopsies from patients with diagnosis of FSGS (*n* = 4) were assessed. Patients with FSGS included 2 women (76 and 49 years old) and 2 men (52 and 57 years old) who underwent biopsy for proteinuria (8, 7.3, 11, and 9.2 g/d), with serum creatinine levels normal or mildly increased (0.9, 0.82, 1.94, and 1.4 mg/dL). Interstitial fibrosis/tubular atrophy was 5%–15%, 10%–20%, about 30%, and 20%–30%, respectively. Global and segmental glomerulosclerosis was 0%/7%, 18%/24%, 30%/54%, and 6%/31%, respectively. Three patients who underwent biopsy for hematuria or low-level proteinuria with minimal histologic abnormalities were used as controls. These included 1 man and 2 women, ages 34, 20, and 38 years, respectively, with normal serum creatinine levels, and no proteinuria in 2 patients and 515 mg/d in the 38-year-old woman. There was no significant tubulointerstitial fibrosis and no segmental glomerulosclerosis, and only the biopsy from the 38-year-old woman showed global glomerulosclerosis (4%). None of the above biopsies showed deposits by immunofluorescence or electron microscopy. The FSGS patients showed extensive foot process effacement by electron microscopy.

#### Human liver biopsies.

Wedge biopsies of the left lateral lobe of the liver were obtained at the time of elective bariatric surgery. Patients with NASH included 2 women (39 and 50 years old) and 3 men (25, 35, and 39 years old), while normal individuals included 3 women (34, 39, and 39 years old) and 2 men (35 and 39 years old). The presence of steatosis, ballooning, inflammation, and fibrosis was determined by a histopathologist. Samples were categorized using the NAS scoring criteria (44): steatosis (0 = <5%; 1 = 5%–33%; 2 = 34%–66%; 3 = >66%), ballooning degeneration (0 = none; 1 = few; 2 = many), lobular inflammation (0 = none; 1 = <2 foci per 200 high-power fields [HPFs]; 2 = 2–4 foci per 200 HPFs; 3 = 4 foci per 200 HPFs), fibrosis (0 = none; 1= zones 1–3; 2 = zone 3 and periportal; 3 = bridging; 4 = cirrhosis).

### Immunofluorescence

Paraffin sections were stained with mouse anti-FUS (sc-47711, Santa Cruz Biotechnology), rabbit anti-GFP (NB600-308, Novus Biological), rabbit anti–phospho-EGFR (Tyr1173; 4407, Cell Signaling Technology), or rabbit anti–collagen IV (600401106, Rockland) antibodies followed by Alexa Fluor 488 anti-mouse (A32723, Alexa Fluor) and Alexa Fluor 555 anti-rabbit (A21428, Alexa Fluor). Slides were then mounted using ProLong Gold Antifade Mountant with DAPI (P36931, Thermo Fisher Scientific).

Images were taken using a Leica DM6000B upright microscope (×20 or ×40 lens) equipped with a Leica EC4 microscope camera, and images were captured using LAS X Widefield Systems software.

For kidneys, the number of FUS-positive cells per glomerulus and the intensity of collagen IV per glomerulus were analyzed using ImageJ (NIH), and values were expressed as percentage of FUS-positive cells among total glomerular cells or collagen IV intensity per glomerulus.

For livers, FUS nuclear intensity was analyzed using CellProfiler, and values were expressed as FUS nuclear intensity per microscopic field. Collagen IV intensity was analyzed as described above and expressed as collagen IV intensity per microscopy field.

### Nonnuclear (cytosolic) and nuclear fractions

Nonnuclear and nuclear tissue fractions were obtained as previously described (11). Kidney cortices or livers (10 mg) were homogenized in 250 mM sucrose, 10 mM HEPES, pH 7.4, 5 mM KCl, 1.5 mM EDTA, pH 8.0, 5 mM Na_3_VO_4_, and protease inhibitors. After 15 minutes on ice, tissue lysates were centrifuged. The resulting pellet was saved as nuclear fraction and resuspended in 20 mM HEPES, pH 7.4, 0.4 M NaCl, 2.5% glycerol, 1 mM EDTA, pH 8.0, 0.5 mM NaF, and protease inhibitors, and the supernatant was saved as nonnuclear fraction.

When cells were used for analysis, they were suspended in 10 mM HEPES, pH 7.9, 1.5 mM MgCl_2_, 10 mM KCl, protease inhibitors (Roche Applied Science), and 5 mM NaVO_3_, and nonnuclear and nuclear fractions were separated by centrifugation (400*g* for 4 minutes at 4°C). Nuclear fractions were lysed in the buffer described above containing 25% glycerol.

### In vitro cell analysis

Murine HSC line JS1 (29) was cultured in DMEM supplemented with 10% FBS. Before experiments, JS1 cells were serum-starved for 24 hours. JS1 cells were treated with EGF (20 ng/mL; 236-EG, R&D Systems) or vehicle alone for various time intervals. Cell lysates as well as nonnuclear and nuclear fractions of treated JS1 cells were analyzed by Western blot analysis. To inhibit FUS nuclear translocation, serum-starved JS1 cells were incubated with CP-FUS-NLS or CP-mutFUS-NLS (both at 0.4 μM). After 30 minutes, cells were treated with vehicle (PBS) or EGF (20 ng/mL). After 0.5 to 24 hours, cells were processed for nonnuclear and nuclear fractionation and Western blot analysis.

### Western blotting

Tissue or cell lysates were resolved in 8% or 4%–20% SDS-PAGE and transferred to nitrocellulose membranes. Membranes were incubated with the following primary antibodies: rabbit anti-FUS/TLS (4885, Cell Signaling Technology), rabbit anti–histone H3 (9715, Cell Signaling Technology), mouse anti–α-tubulin (3873, Cell Signaling Technology), rat anti–collagen IV α2NC1 (7071, Chondrex), rabbit anti–collagen IV (600-401-106, Rockland), rabbit anti–collagen I (66948, Cell Signaling Technology), rabbit anti-desmin (ab15200, Abcam), mouse anti-TIMP1 (sc-s21734, Santa Cruz Biotechnology). IRDye fluorescent dyes (LI-COR Inc.) were used as secondary antibodies, and membranes were processed through a LI-COR Odyssey infrared imaging system. Immunoreactive bands were quantified by densitometry analysis using Image Studio Lite (LI-COR).

### Reverse transcription real-time quantitative PCR

RNA was isolated from tissue with an RNeasy Mini Kit (catalog 74104, Qiagen). cDNA synthesis was performed using 100 ng RNA with an iScript cDNA Synthesis Kit (Bio-Rad), and reverse transcription real-time quantitative PCR was performed with the SYBR Green method using an iQ Real-Time SYBR Green PCR Supermix Kit (Bio-Rad). Fluorescence was acquired at each cycle on a QuantStudio 7 Pro Real-Time PCR System (Thermo Fisher Scientific) using the following cycling conditions: 95°C for 2 minutes, 95°C for 5 seconds, 57°C for 45 seconds, and 60°C for 30 seconds for 40 cycles; and 65°C to 95°C with an increment of 0.5°C in 5 seconds. The quantitation cycle values were analyzed using the QuantStudio 7 Pro Real-Time PCR System and normalized to GAPDH levels. Primers used for mouse *Col4A2* chain, mouse *Col1A2* chain, and *Gapdh* were: *Col4A2* forward, 5′-TCATTAGCAGGTGTGCGGTT-3′; *Col4A2* reverse, 5′-AGCGGGGTGTGTTAGTTACG-3′; *Col1A2* forward, 5′-CTTGCTGGCCTACATGGTGA-3′; *Col1A2* reverse, 5′-ATGAGTTCTTCGCTGGGGTG-3′; *Gapdh* forward, 5′-CATCTTGGGCTACACTGAGG-3′; and *Gapdh* reverse, 5′-GTGGTCCAGGGTTTCTTACTC-3′ (45).

### RNA sequencing and data analysis

The RNA sequencing data of human kidneys (FSGS versus normal) and liver (NASH versus normal) were obtained from the NCBI Gene Expression Omnibus (GEO) database using accession numbers GSE129973 and GSE164760, respectively. The data set GSE129973 contains glomerular transcriptome from human kidney biopsies (https://www.ncbi.nlm.nih.gov/geo/query/acc.cgi?acc=GSE129973). The data set GSE164760 contains NASH samples collected from different institutions (https://www.ncbi.nlm.nih.gov/geo/query/acc.cgi?acc=GSE164760). The analysis was performed using the GEO2R interactive web tool (https://www.ncbi.nlm.nih.gov/geo) that uses DESeq2 (46) to perform differential expression analysis. The statistical analysis was generated by DESeq2, and the Wald test was used to compare 2 groups of samples. Changes between 2 experimental conditions (e.g., FSGS versus normal or NASH versus normal) were expressed as log_2_ fold change, and *P* values were used for statistical significance between 2 groups. A volcano plot for each data set was generated using DESeq2, which displays statistical significance (–log_10_ *P* value) versus magnitude of change (log_2_ fold change).

### Statistics

Data are shown as mean ± SD. Unpaired 2-tailed *t* test was used to evaluate statistically significant differences (*P* < 0.05) between 2 groups. One-way ANOVA followed by Dunnett’s multiple-comparison test, by GraphPad Prism software, was used to evaluate statistically significant differences among multiple groups. Data distribution was assumed to be normal, but this was not formally tested.

### Study approval

#### Mouse studies.

All in vivo experiments were performed with approval of and according to the Vanderbilt University Medical Center Institutional Animal Care and Use Committee guidelines and conducted in Association for Assessment and Accreditation of Laboratory Animal Care–accredited facilities.

#### Human samples.

The use of human archival identified kidney specimens was approved by the IRB of Vanderbilt University (040174 “Renal Pathology”). For liver samples, participants gave informed written consent before participating in this study, which was approved by the IRB of Vanderbilt University (090657 and 171845) and registered at ClinicalTrials.gov (NCT00983463 and NCT03407833). All studies were conducted in accordance with NIH and institutional guidelines for human subject research. The study protocol conformed to the ethical guidelines of the 1975 Declaration of Helsinki, as reflected in a priori approval by Vanderbilt University Medical Center.

### Data availability

Raw data are available in the Supporting Data Values file.

## Author contributions

MC conducted experiments, acquired data, analyzed data, and wrote the first draft of the manuscript. YAL provided reagents and edited the manuscript. MZZ provided reagents. RCH provided reagents and edited the manuscript. TS performed experiments. VL provided mice. CRB provided reagents and edited the manuscript. ABF provided kidney samples and data and edited the manuscript. CRF provided liver samples and data and edited the manuscript. GY provided mice. JZ provided reagents and contributed to the first draft of the manuscript. JH and RZ provided reagents and edited the manuscript. AP designed research studies, analyzed data, and wrote the manuscript.

## Supplementary Material

Supplemental data

Unedited blot and gel images

Supporting data values

## Version 1 03/15/2024

Electronic publication

## References

[1] Henderson NC (2020). Fibrosis: from mechanisms to medicines. Nature.

[2] Borza CM (2022). Genetic and pharmacological tools to study the role of discoidin domain receptors in kidney disease. Front Pharmacol.

[3] Chen X (2004). Lack of integrin α1β1 leads to severe glomerulosclerosis after glomerular injury. Am J Pathol.

[4] Chen X (2007). Integrin α1β1 controls reactive oxygen species synthesis by negatively regulating epidermal growth factor receptor-mediated Rac activation. Mol Cell Biol.

[5] Harris RC (2021). The epidermal growth factor receptor axis and kidney fibrosis. Curr Opin Nephrol Hypertens.

[6] Bollee G (2011). Epidermal growth factor receptor promotes glomerular injury and renal failure in rapidly progressive crescentic glomerulonephritis. Nat Med.

[7] Komuves LG (2000). Expression of epidermal growth factor and its receptor in cirrhotic liver disease. J Histochem Cytochem.

[8] Hoshida Y (2013). Prognostic gene expression signature for patients with hepatitis C-related early-stage cirrhosis. Gastroenterology.

[9] Falleti E (2012). Association between the epidermal growth factor rs4444903 G/G genotype and advanced fibrosis at a young age in chronic hepatitis C. Cytokine.

[10] Petrelli F (2016). Antibiotic prophylaxis for skin toxicity induced by antiepidermal growth factor receptor agents: a systematic review and meta-analysis. Br J Dermatol.

[11] Chiusa M (2020). EGF receptor-mediated FUS phosphorylation promotes its nuclear translocation and fibrotic signaling. J Cell Biol.

[12] Wang G (2021). Fus knockdown inhibits the profibrogenic effect of cardiac fibroblasts induced by angiotensin II through targeting Pax3 thereby regulating TGF-β1/Smad pathway. Bioengineered.

[13] Tsukie T (2014). Decreased amount of collagen in the skin of amyotrophic lateral sclerosis in the Kii Peninsula of Japan. Acta Neurol Taiwan.

[14] Ono S (1999). Decreased urinary concentrations of type IV collagen in amyotrophic lateral sclerosis. Acta Neurol Scand.

[15] Ono S (1998). Decreased type IV collagen of skin and serum in patients with amyotrophic lateral sclerosis. Neurology.

[16] Bryant C (2022). Adriamycin-induced nephropathy is robust in N and modest in J substrain of C57BL/6. Front Cell Dev Biol.

[17] Barrick CJ (2009). Reduced EGFR causes abnormal valvular differentiation leading to calcific aortic stenosis and left ventricular hypertrophy in C57BL/6J but not 129S1/SvImJ mice. Am J Physiol Heart Circ Physiol.

[18] Sephton CF (2014). Activity-dependent FUS dysregulation disrupts synaptic homeostasis. Proc Natl Acad Sci U S A.

[19] Cuttler AS (2011). Characterization of Pdgfrb-Cre transgenic mice reveals reduction of ROSA26 reporter activity in remodeling arteries. Genesis.

[20] He B (2021). Single-cell RNA sequencing reveals the mesangial identity and species diversity of glomerular cell transcriptomes. Nat Commun.

[21] Heikkilä E (2010). β-Catenin mediates adriamycin-induced albuminuria and podocyte injury in adult mouse kidneys. Nephrol Dial Transplant.

[22] Fuchs BC (2014). Epidermal growth factor receptor inhibition attenuates liver fibrosis and development of hepatocellular carcinoma. Hepatology.

[23] Dong S (2016). Mechanisms of CCl4-induced liver fibrosis with combined transcriptomic and proteomic analysis. J Toxicol Sci.

[24] Zhang D (2018). Desmin- and vimentin-mediated hepatic stellate cell-targeting radiotracer ^99m^Tc-GlcNAc-PEI for liver fibrosis imaging with SPECT. Theranostics.

[25] Hoffmann C (2020). Hepatic stellate cell hypertrophy is associated with metabolic liver fibrosis. Sci Rep.

[26] Lee YS, Seki E (2023). In vivo and in vitro models to study liver fibrosis: mechanisms and limitations. Cell Mol Gastroenterol Hepatol.

[27] Liang D (2018). Inhibition of EGFR attenuates fibrosis and stellate cell activation in diet-induced model of nonalcoholic fatty liver disease. Biochim Biophys Acta Mol Basis Dis.

[28] Arabpour M (2014). Targeted elimination of activated hepatic stellate cells by an anti-epidermal growth factor-receptor single chain fragment variable antibody-tumor necrosis factor-related apoptosis-inducing ligand (scFv425-sTRAIL). J Gene Med.

[29] Guo J (2009). Functional linkage of cirrhosis-predictive single nucleotide polymorphisms of Toll-like receptor 4 to hepatic stellate cell responses. Hepatology.

[30] Dormann D, Haass C (2013). Fused in sarcoma (FUS): an oncogene goes awry in neurodegeneration. Mol Cell Neurosci.

[31] Nakaya T, Maragkakis M (2018). Amyotrophic Lateral Sclerosis associated FUS mutation shortens mitochondria and induces neurotoxicity. Sci Rep.

[32] Dormann D (2010). ALS-associated fused in sarcoma (FUS) mutations disrupt Transportin-mediated nuclear import. EMBO J.

[33] Darovic S (2015). Phosphorylation of C-terminal tyrosine residue 526 in FUS impairs its nuclear import. J Cell Sci.

[34] Monjazeb S, Wilson J (2017). Epidermal growth factor receptor inhibitors: cutaneous side effects and their management. Skin Therapy Lett.

[35] Barber NA, Ganti AK (2011). Pulmonary toxicities from targeted therapies: a review. Target Oncol.

[36] Ajmone-Cat MA (2019). Increased FUS levels in astrocytes leads to astrocyte and microglia activation and neuronal death. Sci Rep.

[37] Tavassoly O (2020). Inhibition of brain epidermal growth factor receptor activation: a novel target in neurodegenerative diseases and brain injuries. Mol Pharmacol.

[38] Dhar SK (2014). FUsed in sarcoma is a novel regulator of manganese superoxide dismutase gene transcription. Antioxid Redox Signal.

[39] Awad AM (2020). Erlotinib can halt adenine induced nephrotoxicity in mice through modulating ERK1/2, STAT3, p53 and apoptotic pathways. Sci Rep.

[40] Zahid M, Robbins PD (2015). Cell-type specific penetrating peptides: therapeutic promises and challenges. Molecules.

[41] Nam SH (2023). Recent advances in selective and targeted drug/gene delivery systems using cell-penetrating peptides. Arch Pharm Res.

[42] Luetteke NC (1994). The mouse waved-2 phenotype results from a point mutation in the EGF receptor tyrosine kinase. Genes Dev.

[43] Borza CM (2012). Inhibition of integrin α2β1 ameliorates glomerular injury. J Am Soc Nephrol.

[44] Kleiner DE (2005). Design and validation of a histological scoring system for nonalcoholic fatty liver disease. Hepatology.

[45] Chiusa M (2019). The extracellular matrix receptor discoidin domain receptor 1 regulates collagen transcription by translocating to the nucleus. J Am Soc Nephrol.

[46] Love MI (2014). Moderated estimation of fold change and dispersion for RNA-seq data with DESeq2. Genome Biol.

