# Supplementary material for: Cytoplasmic retention of the DNA/RNA-binding protein FUS ameliorates organ fibrosis in mice
Source: J Clin Invest. 2024 Mar 15;134(6):e175158. doi: 10.1172/JCI175158 (PMC10940094; doi:10.1172/JCI175158)
Supplement: Supplemental data [file jci-134-175158-s161.pdf]

## Supplemental Information

# Cytoplasmic retention of the DNA/RNA binding protein FUS ameliorates organ fibrosis in mice

Manuel Chiusa[1], Youngmin A. Lee[2], Ming-Zhi Zhang[1], Raymond C. Harris[1,3], Taylor Sherrill[4], Volkhard Lindner[5], Craig R. Brooks[1], Gang Yu[6], Agnes B. Fogo[7,1], Charles R. Flynn[2], Jozef Zienkiewicz[8,3], Jacek Hawiger[8,3], Roy Zent[1,3], Ambra Pozzi[1,3]

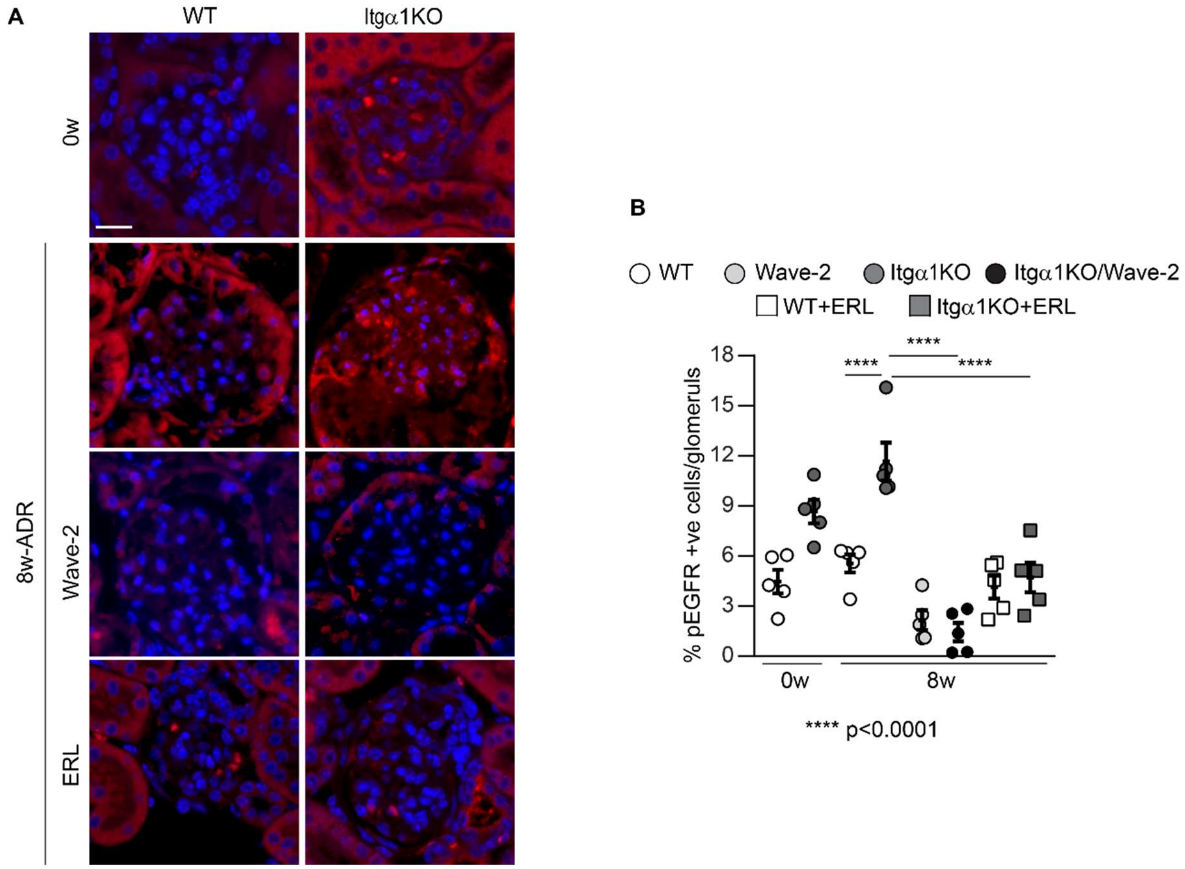


**Supplemental Figure 1**

**Analysis of glomerular phosphorylated EGFR.** (**A**) Representative images of kidney sections from uninjured (0w) or 8-week ADR-treated WT and *ItgA1KO* mice crossed with *Wave2* mice or treated with erlotinib (ERL) stained with anti-phospho EGFR (red) or DAPI (blue). Scale bar, 15 μm. (**B**) Glomerular red fluorescence staining was evaluated using Image J as described in the Methods and expressed as % of phosphorylated EGFR positive cells/glomerulus. Values are the mean ± SD, and symbols represent individual kidneys (n=5 for all genotypes and treatments with an average of at least 10 glomeruli/kidney). Statistical analysis: one-way ANOVA followed by Dunnett's Multiple Comparison Test.

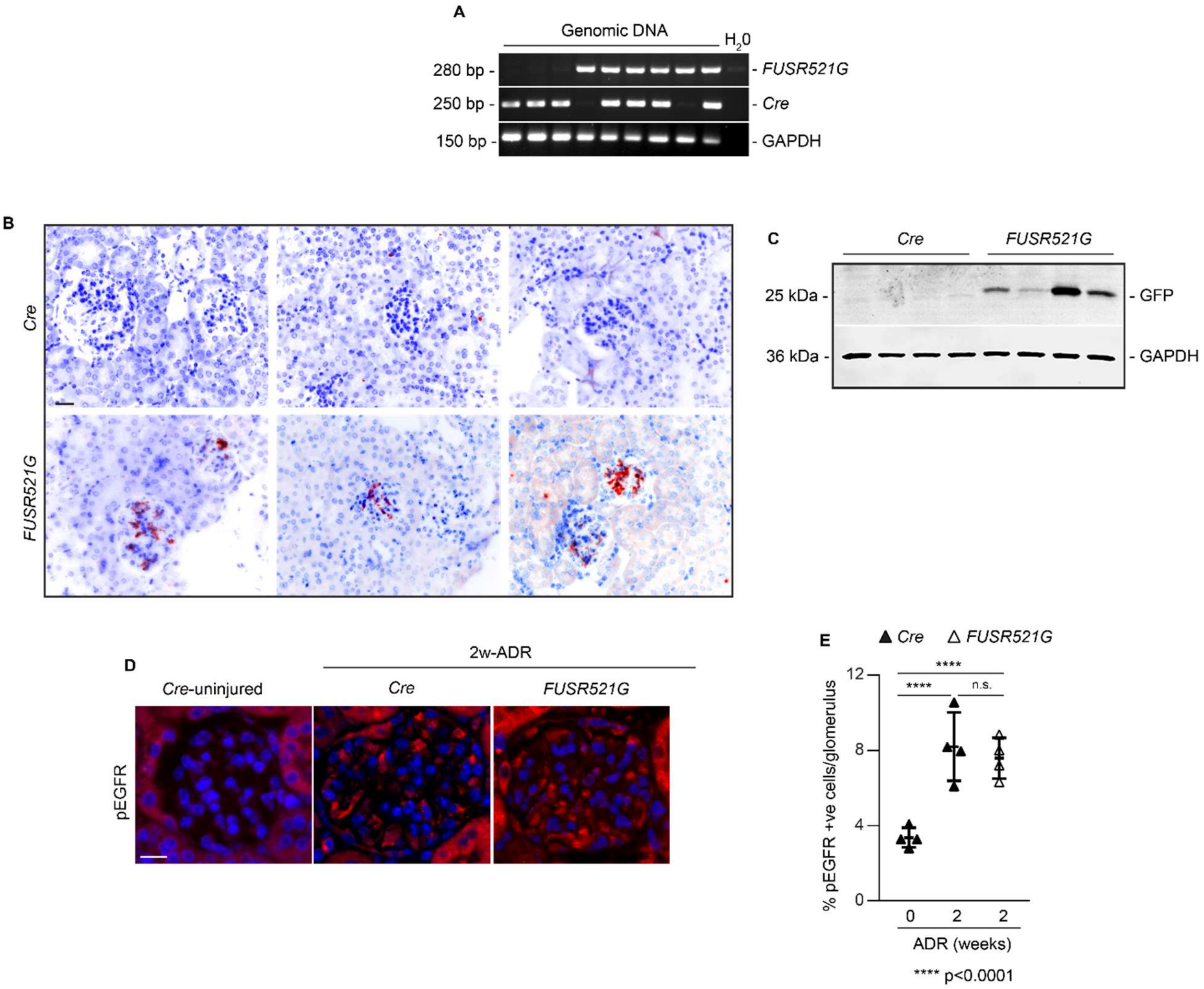


**Supplemental Figure 2**

**Expression and localization of human FUSR521G mutant protein.** (**A**) Genotyping of progeny of *CAG-LacZ$^{fl/fl}$-FUSR521G-IRES-EGFP* mice crossed with *Pdgfrb-cre* mice showing the successful generation of *FUSR521G;Pdgfrb-cre* (*FUSR521G*) positive mice. *FUSR521G* negative for *Cre* or *Cre* mice negative for *FUSR521G* were used as controls for the in vivo experiments performed in this study. (**B, C**) Kidney paraffin sections (**B**) or total kidney lysates (**C**) from control (*Cre* n=4) and *FUSR521G* (n=4) mice were analyzed for GFP localization and expression using selective anti-GFP antibody. Scale bar in (B), 15 µm. (**D**) Representative images of kidney sections from uninjured (*Cre*) or 2-week-ADR-treated *Cre* and *FUSR521G;Pdgfrb-cre* (*FUSR521G*) mice stained with anti-phospho EGFR (red) or DAPI (blue). Scale bar, 15 µm. (**E**) Glomerular red fluorescence staining was evaluated using Image J as described in the Methods and expressed as % of phosphorylated EGFR positive cells/glomerulus. Values are the mean ± SD, and symbols represent individual kidneys (*Cre*-0w n=4, *Cre*-2w n=4, *FUSR521G*-2w n=4 with an average of at least 10 glomeruli/kidney). Statistical analysis: one-way ANOVA followed by Dunnett's Multiple Comparison Test (B, D, E).

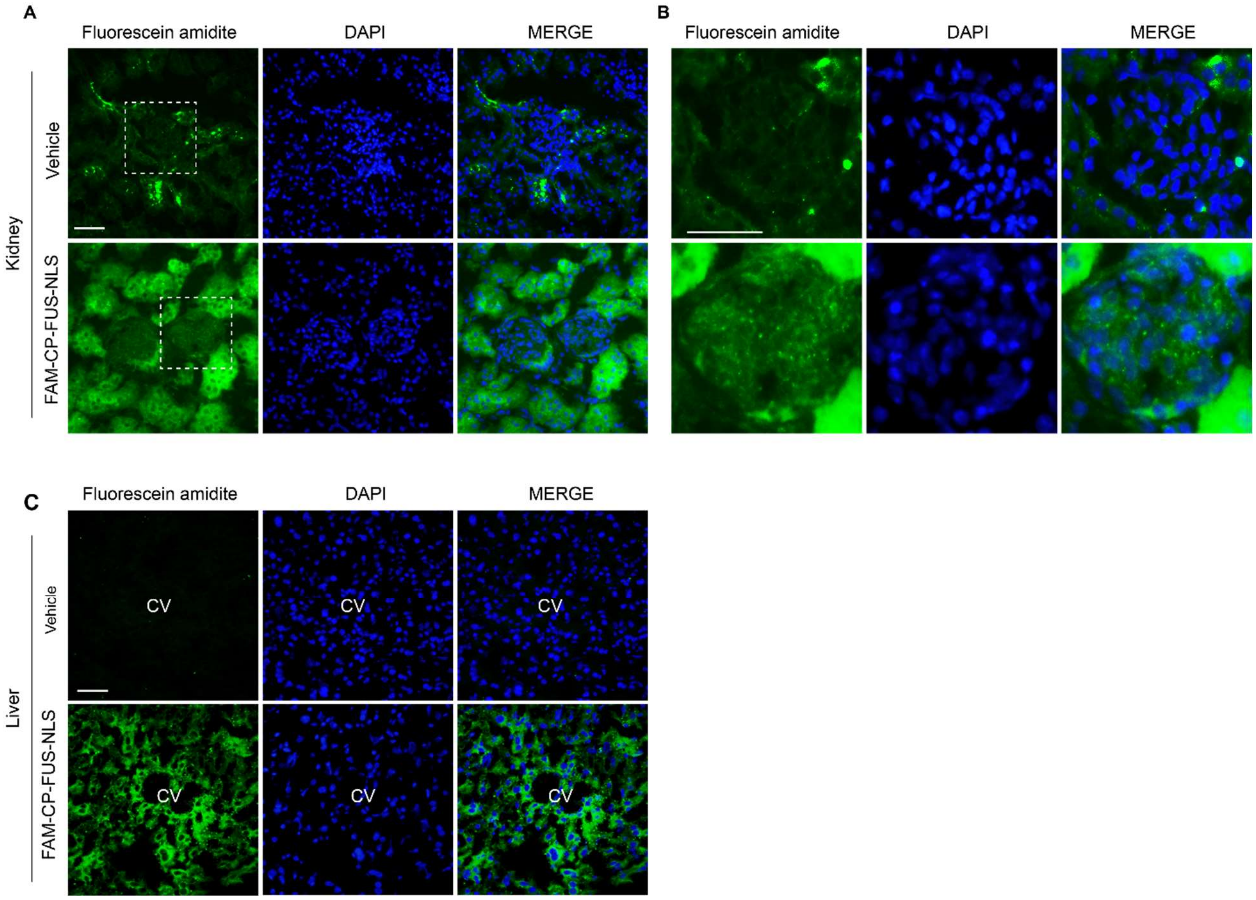


**Supplemental Figure 3**

**Localization of CP-FUS-NLS peptide in kidney and liver.** C57BL/6 J male mice received i.p. injections of vehicle (PBS) or fluorescein amidite (FAM)-conjugated CP-FUS-NLS every two hours for a total of 6 hours. Two hours after the last injection, kidneys and livers were collected. (**A, B**) Representative green fluorescence images of frozen sections of kidneys collected from the mice described above. Note the presence FAM-CP-FUS-NLS peptide in both tubules and glomeruli. Scale bar, 25 µm. (**C**) Representative green fluorescence images of frozen sections of livers collected from the mice described above. Positive green staining was detected in the livers of FAM-CP-FUS-NLS-injected mice. CV, central vein. Scale bar, 25 µm.

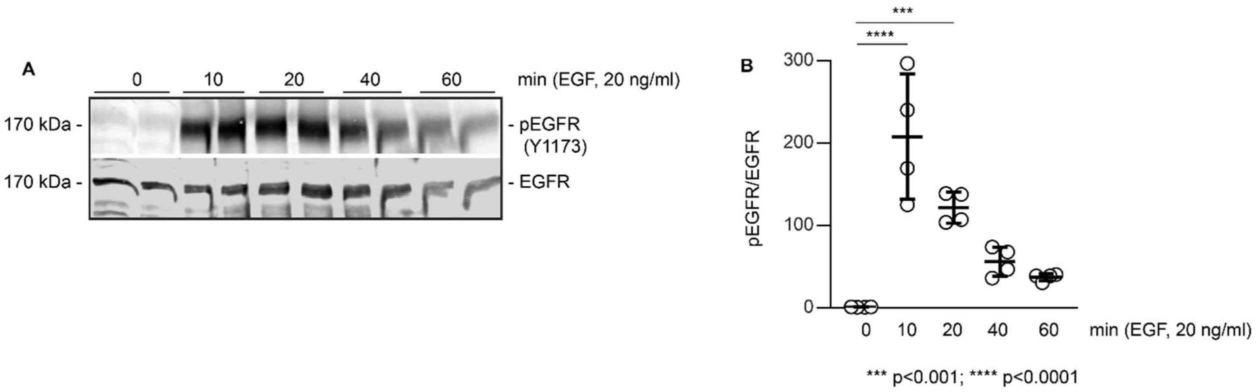


**Supplemental Figure 4**

**Murine HSC line J1 responds to EGF treatment.** (**A**) Total cell lysate (50 µg/lane) of serum-starved J1 cells treated for 1-60 min with EGF (20 ng/ml) were analyzed by Western blot for levels of phosphorylated and total EGFR. (**B**) Bands were quantified by densitometry analysis and values represent pEGFR/EGFR ratio. Values are the mean ± SD, and symbols represent individual experiments (n=5). Statistical analysis: one-way ANOVA followed by Dunnett's Multiple Comparison Test.
