# Supplementary material for: Cytoplasmic retention of the DNA/RNA-binding protein FUS ameliorates organ fibrosis in mice
Source: J Clin Invest. 2024 Mar 15;134(6):e175158. doi: 10.1172/JCI175158 (PMC10940094; doi:10.1172/JCI175158)

Figure 5

A

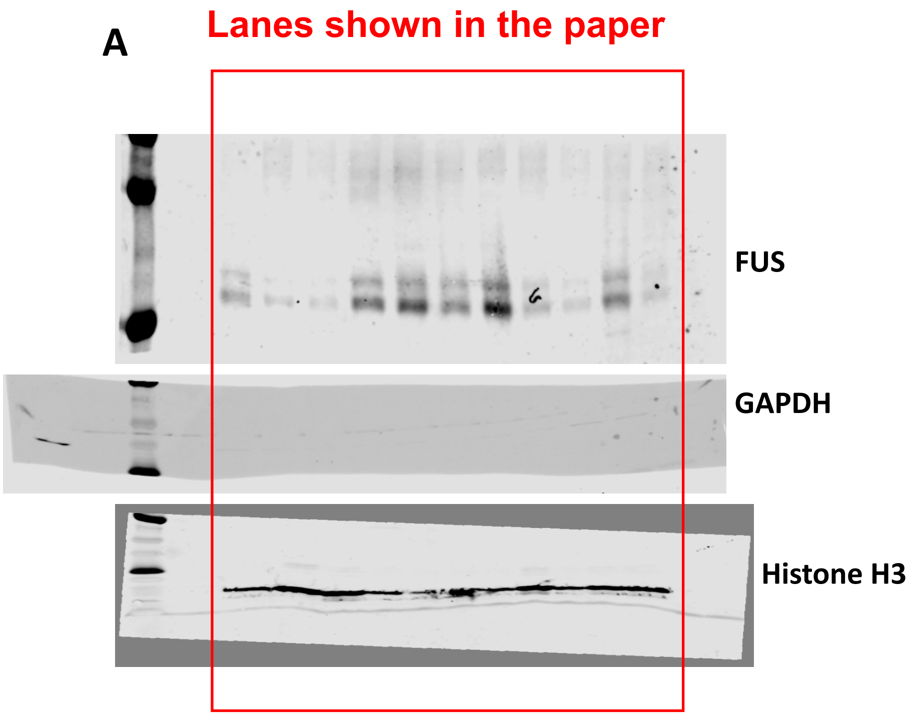

Figure 8

A

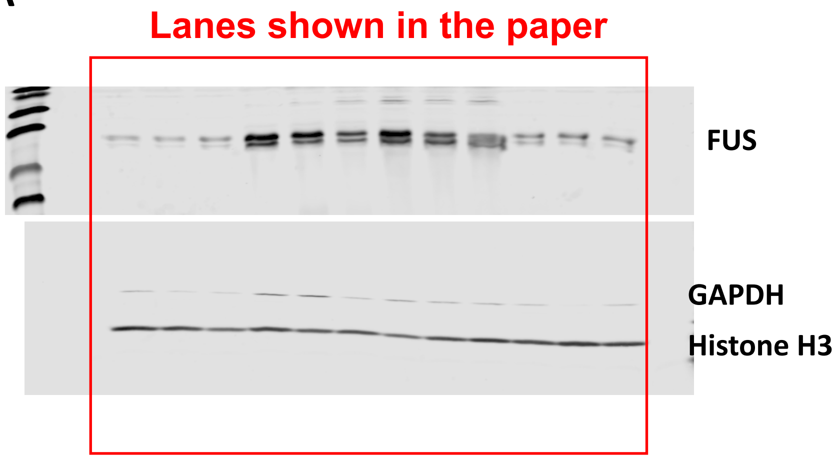
Lanes shown in the paper
FUS
GAPDH
Histone H3

B

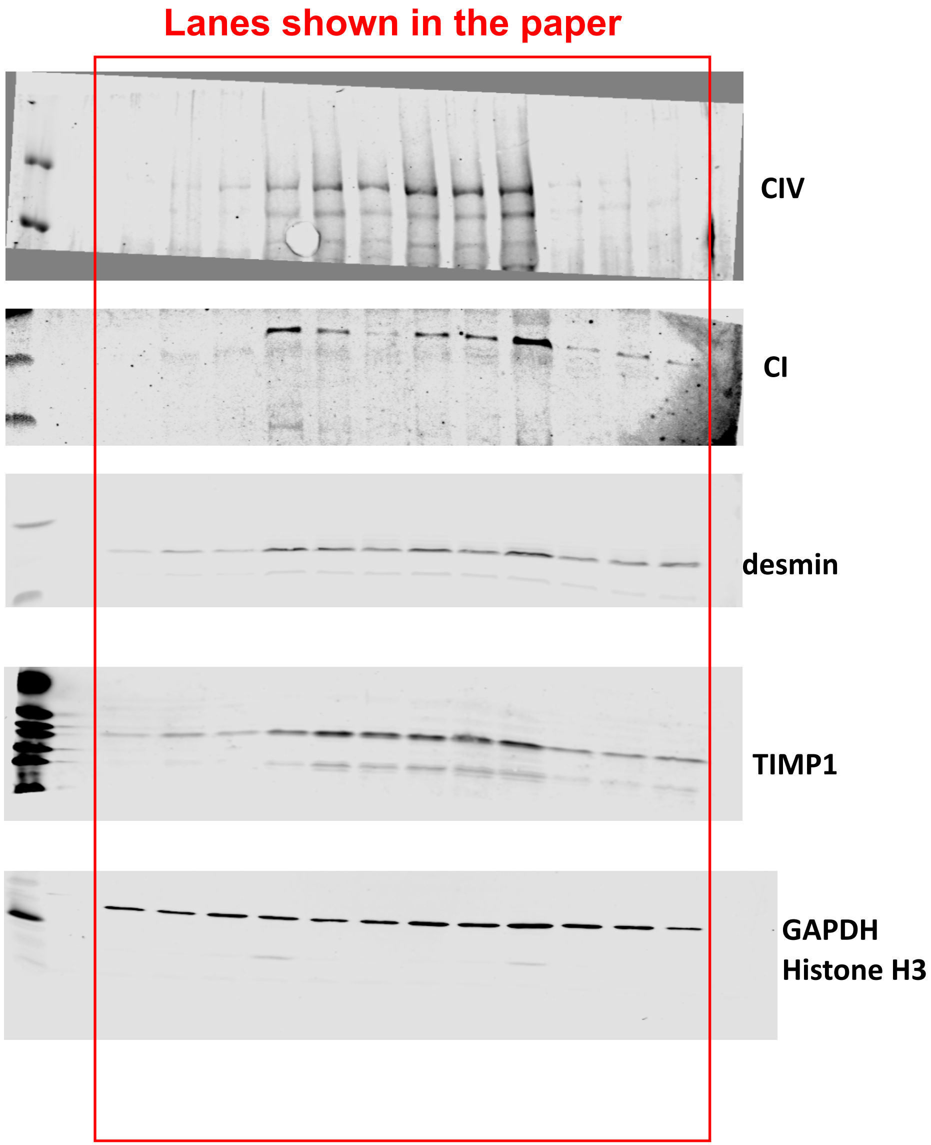
Lanes shown in the paper
CIV
CI
desmin
TIMP1
GAPDH
Histone H3

Figure 9

A

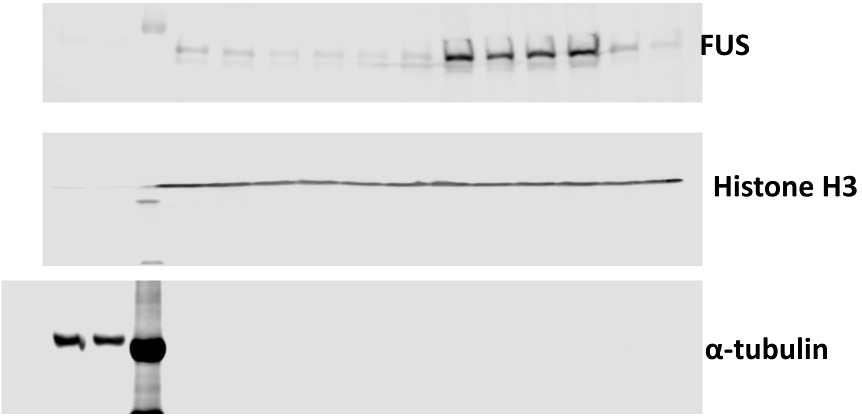

C

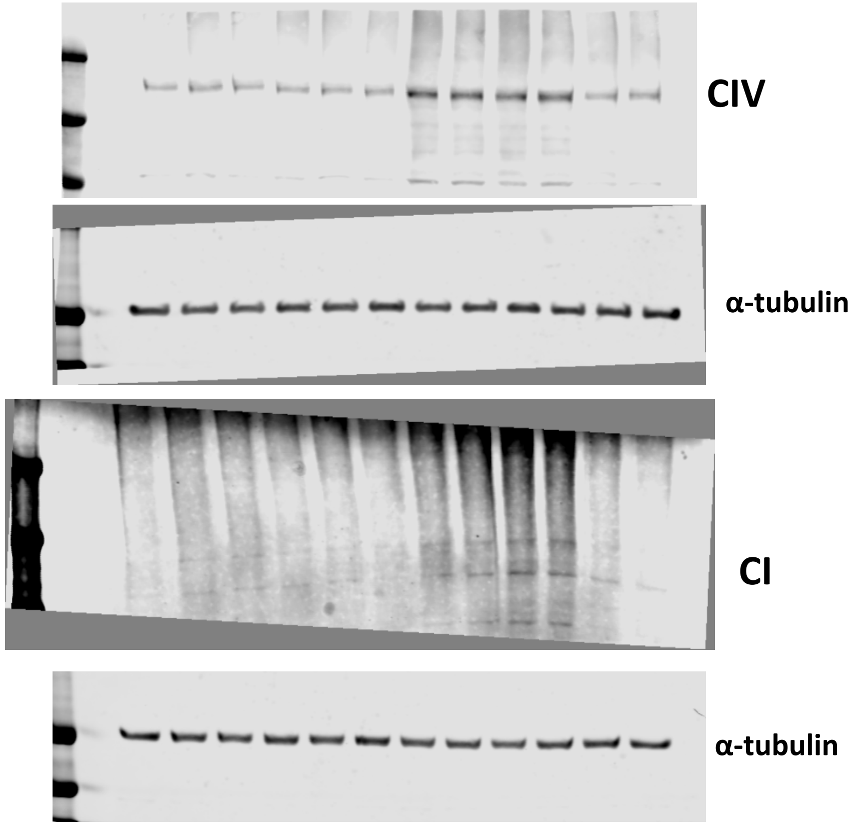

Supplemental Fig. 2

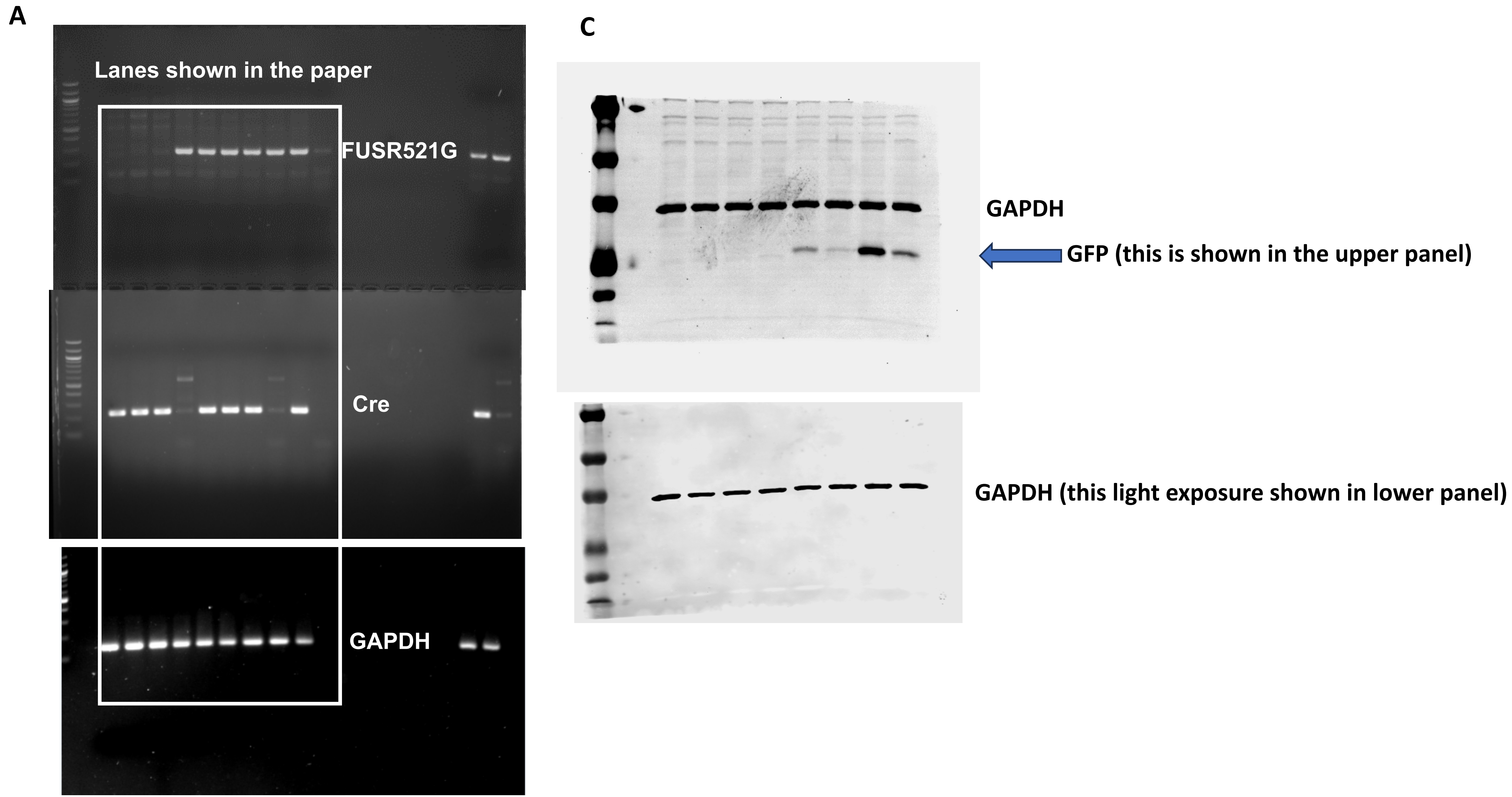

Supplemental Fig. 4

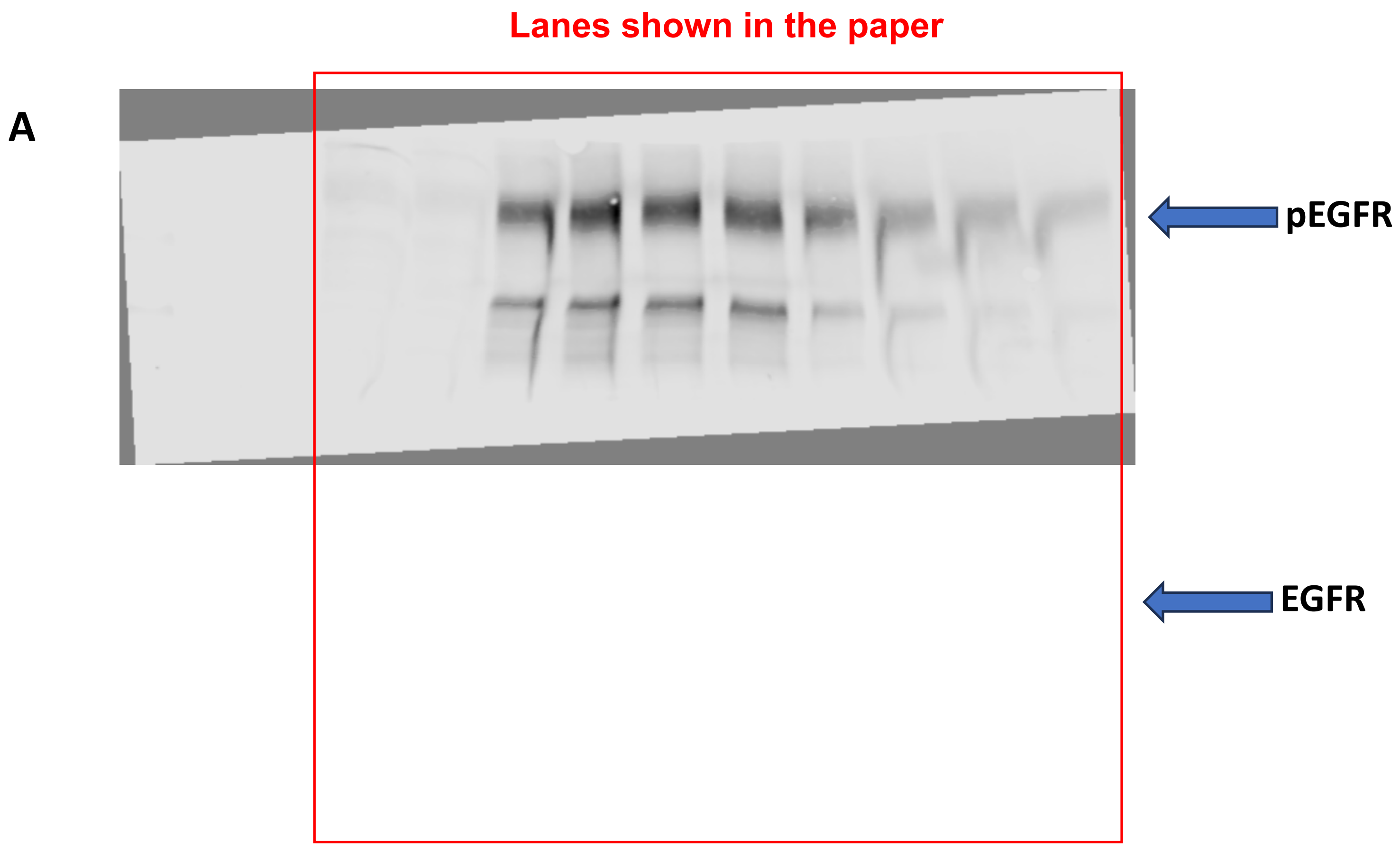

Supplement: Unedited blot and gel images [file jci-134-175158-s162.pdf]
